# Next-generation sequencing of a combinatorial peptide phage library screened against ubiquitin identifies peptide aptamers that can inhibit the *in vitro* ubiquitin transfer cascade

**DOI:** 10.3389/fmicb.2022.875556

**Published:** 2022-12-02

**Authors:** Małgorzata Lisowska, Fiona Lickiss, Maria Gil-Mir, Anne-Sophie Huart, Zuzanna Trybala, Luke Way, Lenka Hernychova, Adam Krejci, Petr Muller, Radovan Krejcir, Igor Zhukow, Przemyslaw Jurczak, Sylwia Rodziewicz-Motowidło, Kathryn Ball, Borivoj Vojtesek, Ted Hupp, Umesh Kalathiya

**Affiliations:** ^1^International Centre for Cancer Vaccine Science, University of Gdańsk, Gdańsk, Poland; ^2^University of Edinburgh, Institute of Genetics and Molecular Medicine, Edinburgh, United Kingdom; ^3^Research Centre for Applied Molecular Oncology, Masaryk Memorial Cancer Institute, Brno, Czechia; ^4^Institute of Biochemistry and Biophysics, Polish Academy of Sciences, Warsaw, Poland; ^5^Faculty of Chemistry, University of Gdańsk, Gdańsk, Poland

**Keywords:** ubiquitin, phage-peptide, next-generation sequencing, aptamers, molecular dynamics, protein–peptide binding

## Abstract

Defining dynamic protein–protein interactions in the ubiquitin conjugation reaction is a challenging research area. Generating peptide aptamers that target components such as ubiquitin itself, E1, E2, or E3 could provide tools to dissect novel features of the enzymatic cascade. Next-generation deep sequencing platforms were used to identify peptide sequences isolated from phage-peptide libraries screened against Ubiquitin and its ortholog NEDD8. In over three rounds of selection under differing wash criteria, over 13,000 peptides were acquired targeting ubiquitin, while over 10,000 peptides were selected against NEDD8. The overlap in peptides against these two proteins was less than 5% suggesting a high degree in specificity of Ubiquitin or NEDD8 toward linear peptide motifs. Two of these ubiquitin-binding peptides were identified that inhibit both E3 ubiquitin ligases MDM2 and CHIP. NMR analysis highlighted distinct modes of binding of the two different peptide aptamers. These data highlight the utility of using next-generation sequencing of combinatorial phage-peptide libraries to isolate peptide aptamers toward a protein target that can be used as a chemical tool in a complex multi-enzyme reaction.

## Introduction

The ubiquitin conjugation system has emerged as a compelling landscape in the drug discovery field (Gong et al., 2010) with PROTAC technology as an example of an innovative synthetic tool (Hu and Crews, 2021). Ubiquitin and its orthologs NEDD8, SUMO, and ISG15 are major post-translational adaptors that target proteins for a variety of molecular fates including protein degradation or stabilization, intracellular localization *via* trafficking, and/or altered biochemical function by direct or allosteric mechanisms (Ciechanover, 2015). The altered wiring of the ubiquitin and ubiquitin-like (UBL) conjugation network in age-related diseases like Alzheimer’s and cancer highlights the importance in developing tools, technologies, and drug screens to intervene therapeutically in diseases involving proteostasis and proteotoxicity (Labbadia and Morimoto, 2015).

The ubiquitin and UBL conjugation reaction is catalyzed by a multi-enzyme cascade that transfers the ubiquitin or UBL molecule from an initial priming enzyme named E1, through a cascade of transfer molecules including those named E2, E3, and sometimes an E4 that ensures specific covalent linkage to the substrate molecule (Scheffner and Kumar, 2014). The E1 represents the least diverse set of priming orthologs in this conjugation system, in which there is estimated to be approximately four that charge UBL conjugation in different signaling events. These different ubiquitin like molecules include viral infection that utilizes ISG15 (Bialas et al., 2015; Ketscher and Knobeloch, 2015); cytokine signaling that utilizes FAT10, and additional UBLs named SUMO or NEDD8 (Heride et al., 2014). The E2 family of UBL conjugation system is more diverse than the E1 family and is thought to provide a degree of diversity and specificity to more localized proteomes (Middleton et al., 2014). The E3 class is the most diverse group of adaptor molecules that interacts with the E2 to transfer ubiquitin molecules directly to substrate (Das et al., 2013; Metzger et al., 2014).

Small molecule discovery in the ubiquitin system has centered on targeting E1, E2, and E3 ubiquitin (like) conjugation enzymes. The most well-developed examples include ligands that target the E1 enzyme that catalyzes NEDD8 conjugation (Bailly et al., 2016), the E2 ubiquitin conjugation enzyme CDC34 (Ceccarelli et al., 2011), and the peptide-binding pocket of the E3 ubiquitin-NEDD8-SUMO ligase MDM2 (Vassilev et al., 2004). The ubiquitin conjugation machine functions as a dynamic multi-enzyme system and provides multiple contacts for both allosteric control and alterations in protein–protein contacts (Das et al., 2013). The Cullin and MDM2 E3 ubiquitin ligases have formed a model system to identify dynamic allosteric stages in E3-mediated substrate docking and ubiquitination (Wallace et al., 2006; Deshaies and Joazeiro, 2009; Wawrzynow et al., 2009; Robson et al., 2012; Fraser et al., 2015). As these molecular machineries provide a model to study the dynamics of protein–protein interactions in an enzyme conjugation system, there is a need for novel strategies to study dynamics of protein–protein interactions, and reaction mechanisms and identify potential approaches for drug discovery.

Discovery of protein–protein interactions is a fundamental goal in the life sciences (Morelli and Hupp, 2012). The existence of millions of potential interfaces driven by linear motifs provides a large interaction landscape requiring novel tools to dissect signaling mechanisms (Tompa et al., 2014). An example is the use of ribosome display that can select for peptides binding to a target from a combinatorial pool (Fuchs et al., 2013). We have set up a robust next-generation peptide-phage library screening assay to identify peptide aptamers that could be used as tools to dissect ubiquitin reaction mechanisms. The peptide libraries are derived from bacteriophage-peptide combinatorial libraries (Smith, 1985) that are screened against a target antigen. Iterative cycles of screening and amplification can select for peptides that bind with a high specificity for a target protein and has been used previously on the E3 ubiquitin ligase MDM2 (Bottger et al., 1996; Burch et al., 2004). This method is labor intensive and always limited to an analysis of a maximum of dozens of phage clones, analyzed usually one by one. Thus, a limitation of the approach methodologically has been the low sequencing throughout of individual selected bacteriophage. Here, we exploit next-generation sequencing methodologies to deep sequence bacteriophage-peptide pools selected against Ubiquitin. With the advance of next-generation sequencing, it is now possible to sequence thousands of inserts in parallel (Dias-Neto et al., 2009; Derda et al., 2010, 2011), which we now use to identify specific peptides that bind to a target protein (ubiquitin). This methodological approach can be used as a template for dissecting many complex protein–protein interactions with an impact on the proteomic research field.

## Materials and equipment

The major tools used in the data acquisition are listed below:

Ph.D. Phage Display Library−12 (New England Biolabs).

Nickel-coated ELISA 96-well plate (Thermo Scientific).

Roche454 Junior sequencing platform.

Varian Inova 500 NMR spectrometer.

## Materials and methods

### Peptide phage display

Peptide phage display was carried out with Ph.D. Phage Display Library−12 (New England Biolabs). NEB provides an extensive and well-explained instruction manual therefore only changes in panning procedure are described here. Random 12-mer peptides are fused to a minor coat protein (pIII) of M13 phage with pentavalent display on each phage. Displayed peptides are expressed at the N-terminus of the coding region of wild-type pIII and separated by a short spacer (Gly–Gly–Gly–Ser). The complexity of the libraries was defined as 10^9^ independent clones and the titer was about 10^13^ pfu/ml. To avoid wild-type phage contamination, all the solutions were either sterilized by filtration or autoclave.

### Panning procedure

A nickel-coated ELISA 96-well plate (Thermo Scientific) was washed with TBST (TBS 1× (50 mM Tris pH 7.5, 150 mM NaCl)—0.1% (*v*/*v*) Tween-20) then His-tag ubiquitin (1 μg) or NEDD8 (1 μg; ENZO Life Sciences) in TBS was captured for 1 h at room temperature (RT). After 5 washes with TBST, the wells were then incubated for 1 h rocking with 10^11^ phages in TBST. These phages were pre-incubated rocking for 1 h at RT in an empty well. After 10 quick washes (1 min or low stringency) or 5 long washes (25 min or high stringency) with TBST, the phage particles were eluted by incubation with 100 μl of 0.2 M Glycine pH 2.2, 1 mg/ml of BSA, with gentle rocking for 10 min, and neutralized with 15 μl of 1 M Tris pH 9.1. Part of the eluted (not amplified) phages were stored at 4°C for up to 1 week. The majority of the eluted phages (90%) were then amplified by infection of ER2378 cells and the phage particles were precipitated. The biopanning procedure was repeated two times and approximately 10^11^ pfu of the first or second round amplified eluate were used as input phage. In addition, the concentration of Tween-20 in buffers was increased to 0.3% (*v*/*v*) in the second round and to 0.5% in the final round of biopanning.

### PCR on phage

**Figure 1:**
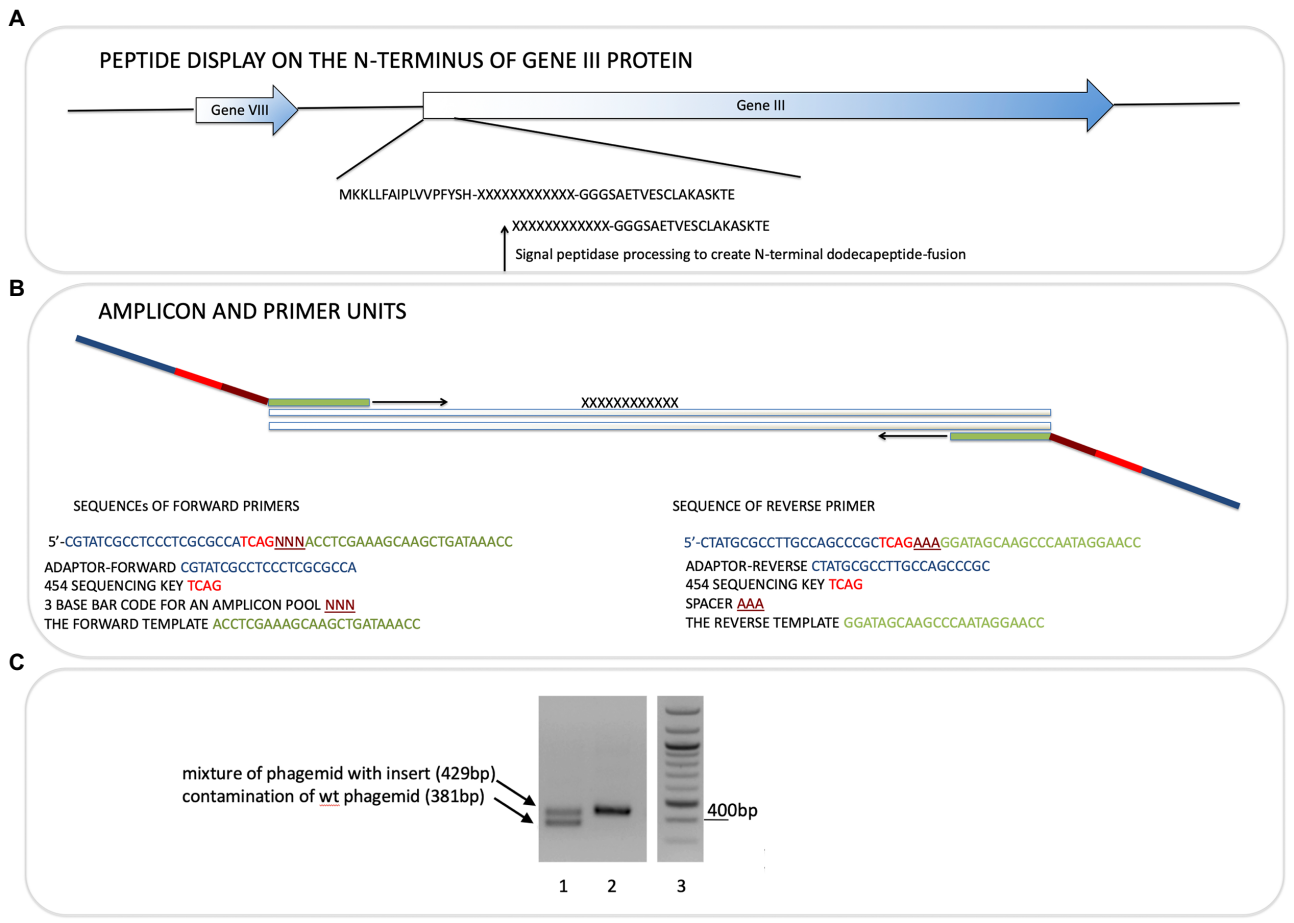
Next-generation sequencing of peptide-phage pools. His-tagged Ubiquitin or NEDD8 proteins were captured onto a nickel coated solid phase. After selection and elution of peptide phage, the phage DNA was amplified using PCR primer sets that capture the sequences flanking the peptide insert. **(A)**. The peptide sequence is at the N-terminus of the gIII M13 coat protein so that the dodecapeptide (X_12_) would have a free N-terminus after signal peptide cleavage and the C-terminus of the peptide is linked to a GGG linker fused to gIII protein (Gene III). **(B)**. The primers used and bar codes for each PCR amplicon are highlighted, and include phages that were amplified or not amplified prior to DNA isolation for sequencing (Table 1). Pooling of all phage into deep sequencing reactions can be done with subsequent deconvolution using the trinucleotide “bar code” (NNN) whose position in the primer is indicated (Supplementary Table 2A; Figure 4 show examples of bar code stratification of peptide counts). **(C)**. An example of the amplification of peptide-phage library pools with primer pairs. The figure highlights a phage pool with a mixture of WT and peptide-containing phage (lane 1), relative to a representative peptide pool containing only peptide insert phage (lane 2) and DNA molecular mass markers (lane 3).

Phage-eluted (not amplified) or amplified phages were used directly in PCR reactions. Primers (Sigma Genosys) used were designed with adaptors for Roche 454 sequencing and are shown in Figure 1. Forward primers were ordered as PAGE purified and reverse primers were HPLC purified. Each sequencing read starts from the amplicon bar code (Figure 1). The insert target sequence length is 36 bp for 12-mer library. The size of the PCR amplicon is therefore 429 bp. Wild-type phage contamination would generate a 381 bp amplicon (see Figure 1). PCR reactions were set up on ice in nuclease-free tubes with final concentrations 1x Herculase II Fusion DNA polymerase buffer (supplied with polymerase), 0.5 M Betain solution for PCR (Sigma), 13.2 mM Trehalose, 500 μM dNTP, 500 nM of each forward and reverse primers, 1 to 10 μl of eluted or amplified phage sample, 1 μl of Herculase II Fusion DNA polymerase (Agilent Technologies), nuclease-free water up to 50 μl. Thermal cycling conditions were: 95°C for 1 min; 95°C for 15 s, 55°C for 20 s, 70°C for 1 min, repeated 30x; 70°C for 3.5 min.

### DNA purification and quantification

The entire PCR reaction was loaded on a 2% (*w*/*v*) agarose gel. Amplicons at the correct size were extracted and purified from 2% (*w*/*v*) agarose gel bands by using the QIAquick Gel Extraction Kit (Qiagen) according to the manufacturer’s instructions including the isopropanol addition step and additional wash with QG buffer. Purified DNA was eluted in 40 μl nuclease-free water. DNA concentration was accurately quantified based on an ultrasensitive fluorescent nucleic acid stain for double-stranded DNA in solution using Quant-iT PicoGreen dsDNA kit (Life Technologies) following the manufacturer’s instructions. Equal quantities of each amplicon sample were pooled together in order to prepare 100 ng (Supplementary Table 1). Exact pool concentration was re-assessed with PicoGreen.

Next,-generation sequencing and DNA sequencing data extraction. Pooled amplicons from each sample were processed by capturing on beads, amplifications using emulsion PCR, and sequenced using the Roche454 Junior according to the manufacturer’s protocols. Data were extracted in Excel using a program made in java (java SE platform) in which each sequence was associated with its iteration number. The program reads .fasta and .qual files simultaneously and for each sequence, the following steps are performed: (1) Length filtering: sequences too short to contain the 36-nucleotide variable region are discarded; (2) The beginning of the read is searched for an exact match with small subsequences of forward and reverse primers. If there is a match, we know this sequence is a forward/reverse read. The bar code is then extracted. If there is no match or the bar code is incomplete, the read is discarded; (3) The 36-nucleotide insert of the read is searched for exact matches with short sequences that should be located at the borders of the variable region. The insert sequence is extracted. If this was reverse read, this sequence is now transformed in reverse and complementary form; (4) If this sequence has 36 nucleotides, it is validated to confirm that all codons are correct (as some codons are not permitted, according to the Ph.D. library manual, there also should be no stop codons). If the sequence has less than 36 nucleotides, it is discarded. If it has more than 36 nucleotides, the program attempts a repair by checking the .qual file and discarding nucleotides of the lowest quality until there are only 36 nucleotides left. Then the program performs a codon check; (5) If a sequence passes the codon check, it is translated; 6) Translated sequences are listed in a table and exported in .csv format. Sequences are then processed, using Macros in Excel (Supplementary Table 2A). To remove existing biases, each sequence that is observed more than twice in the peptide library is removed in all the samples, as they could reflect phage amplification advantage in bacteria. Moreover, all the sequences containing a minimal number of HHH motifs are discarded so as to remove phage binding to nickel-coated plates. Next, data were sorted into tables (Supplementary Tables 2B,C). A short list of synthetic peptides was ordered from Chiron Mimotopes (Australia; Supplementary Table 3) with either format: (i) PEPTIDE-three amino acid space GSG-Lys(Biotin)-amide or an N-terminal biotin is added instead of a C-terminal biotin to test activity with an N-terminal tag, (ii) biotin-conjugated to a four amino acid SGSG spacer followed by a 15-amino acid sequence with a C-terminal amide.

### Peptide-binding assays

**Figure 2:**
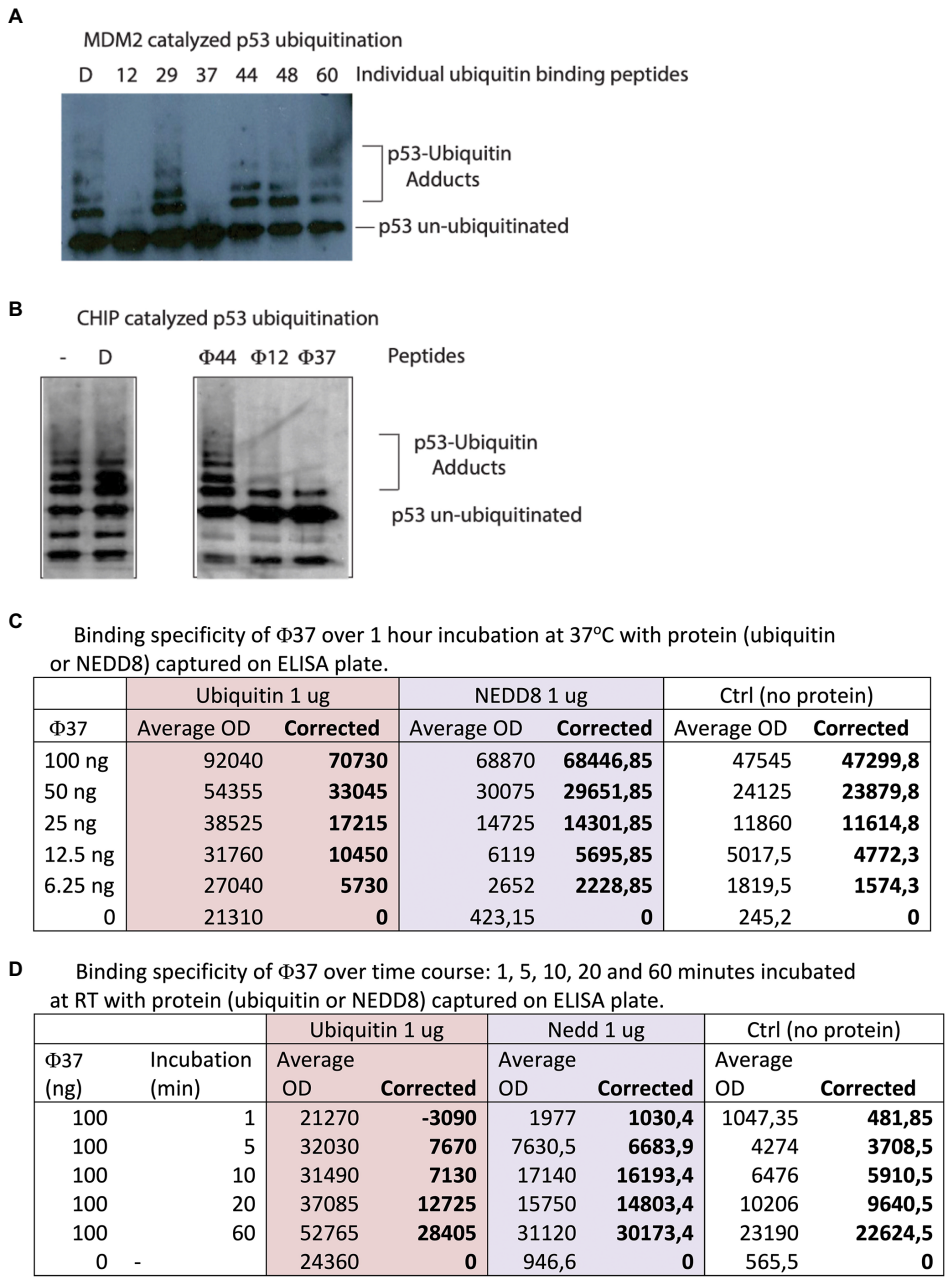
Identification of peptides with bioactivity in ubiquitin assays. **(A)** Ubiquitin assays driven by the E3 ubiquitin ligase MDM2 were assembled with individual peptides from the reactive pools derived from the 60 peptides in Supplementary Table 3. Representative assay showing that specific peptides such as 12 and 37 reproducibly inhibited MDM2 catalyzed ubiquitination (from the left, lanes 2 and 4 vs. lane 1 DMSO-only control). **(B)** Ubiquitin assays driven by the E3 ubiquitin ligase CHIP were assembled with individual peptides (labeled Φ) that were active in inhibiting MDM2-driven reactions (12 and 37) and a non-active ubiquitin-binding peptide 44 (Figure 2B). D represents DMSO-only control. Reactions were processed by immunoblotting to detect changes in p53 ubiquitination as indicated in the methods. **(C,D)** Analysis by ELISA of peptide-37 activity with a C-terminal biotin tag. Ubiquitin or NEDD8 was coated on the solid-phase to mimic the original peptide-phage screen. Peptides were added and then either titrated in duplicates as indicated from 6.25–100 ng for 60 min **(C)** or else fixed peptide levels (100 ng) were added in duplicates as a time course from 1 to 60 min **(D)**. The data are tabulated as binding in ECL units.

Peptide-binding ELISA was carried out in a white 96-well microtiter plate (Costar), which was coated with 1 μg of ubiquitin or 1 μg of NEDD8 (Bio-techne R&D systems) and incubated overnight at 4°C. The plate was blocked using BSA 3% (Sigma) for 1 h at RT. From 0-100 ng of biotinylated peptide-37 (that is H-FIPAQLHFHWRSGSG(LysBiotin) -NH2, in the format Amine-PEPTIDE-GSG-Lys(Biotin)-amide) was added and incubated for 1 h at 37°C (Figure 2C). In another set of experiments, 100 ng of peptides was incubated for 1, 5, 20, and 60min at 37°C (Figure 2D). Binding was detected using streptavidin-HRP-conjugated protein coupled to chemiluminescence on Varioskan Lux Reader (Thermo Fisher Scientific).

### *In vitro* ubiquitination assay

Ubiquitination reaction had the following components: 25 mM HEPES pH 8, 6 mM MgCl2, 0.05% (*v*/*v*) Triton X-100, 3 mM ATP, 0.5 mM DTT, 1 mM benzamidine, 10 μM Ubiquitin (Boston Biochem), 100 nM E1 (UBE1, Boston Biochem), 1 μM E2 (His-Ubch5a, Boston Biochem), 30 μM creatine phosphate, 1.2 μM creatine kinase), 46 nM p53 (50 ng), 37 nM MDM2 (50 ng), the RING domain of MDM2 (0.55 μM, 100 ng), or his-tagged CHIP (12 nM, 10 ng) and the indicated amount of synthetic peptide [1.25 μg (or approximately 25–35 μM of each peptide depending upon precise mass), unless otherwise indicated], and DMSO control to a final concentration that matches the volume of the peptide added (peptides are resuspended in 100% DMSO). The synthetic peptides (from Chiron Mimotopes, Australia) had a N-terminal biotin followed by an SGSG spacer, then the peptide sequence containing a C-terminal amide (See Supplementary Table 3). The MDM2- or CHIP-dependent ubiquitination reactions were carried out at 30°C for 12 min, as described previously (Fraser et al., 2015; Narayan et al., 2015). To stop the reaction 22 μl of 2× sample buffer (5% (*w*/*v*) SDS, 25% (*v*/*v*) glycerol, 200 mM DTT, 0.3M Tris, pH 6.8) was added and the samples were analyzed by 4–12% NuPAGE Gels followed by immunoblotting using the p53 antibody DO1 antibody to measure p53 ubiquitination.

### C18-reverse phase chromatography and deuterium exchange mass spectrometry

The ubiquitin protein either free or in complex with peptide-12 (Biotin-SGSG-AKFDMHIATRLS-amide) or peptide-37 (Biotin-SGSG-FIPAQLHFHWRS-amide) used in Ubiquitination reactions were initiated by dilution into deuterated water with 0.1% DMSO to a final concentration of 2 μM. The molar ratios between ubiquitin and peptide-12 or peptide-37 were 1:2 and 1:5. The incubation was carried out at room temperature and was quenched by the addition of 0.875MHCl in 1 M glycine at 1 min, followed by rapid freezing in liquid nitrogen. Each sample prepared for global HDX-MS analysis was thawed and injected onto trap column and desalted on-line on a microtrap (Michrom Bioresources, Auburn, CA) for 2 min at flow rate 20 μl/min. Next, the protein was eluted onto an analytical column (Jupiter C18, 1.0 × 50 mm, 5 μm, 300 Å, Phenomenex, CA) and separated using a linear gradient elution of 10%B in 2 min, followed by 31 min isocratic elution at 40%B. Solvents were: A, 0.1% formic acid in water; B, 80% acetonitrile/0.08% formic acid. The trap cartridge and the analytical column were kept at 1°C. Mass spectrometric analysis was carried out using an Orbitrap Elite mass spectrometer (Thermo Fisher Scientific) with ESI ionization on-line connected with a robotic system based on the HTS-XT platform (CTC Analytics, Zwingen, Switzerland). Analysis of deuterated samples was done in HPLC-MS mode with ion detection in the orbital ion trap, the data were processed manually, and deconvolution was done with *MagTran* software (Zhang and Marshall, 1998).

### NMR analysis of the ubiquitin-binding peptides

Nuclear magnetic resonance (NMR) measurements were performed with a Varian Inova 500 NMR spectrometer operating at 11.7 T (^1^H resonance frequency 500.606 MHz) equipped with a triple-resonance ^1^H/^13^C/^15^N probe head and z-gradient Performa IV unit. N^15^-labeled Ubiquitin was purchased from Asla Biotech (Latvia). The ^1^H-^15^N HSQC spectra were recorded at 298 K on the sample containing 0.5 mM uniformly labeled ^15^N-human ubiquitin in 10 mM sodium phosphate buffer (92%/8% H_2_O/D_2_O) at pH 5.0 in the absence and presence of the peptides. Non-tagged peptide-12 (AKFDMHIATRLS) or Peptide-37 (FIPAQLHFHWRS) in this case were non-biotinylated with a C-terminal NH_2_. The reason a C-terminal amide was used instead of a carboxylic acid is that the original peptide when fused to gIII protein in the phage would not have an acidic C-terminus. The ubiquitin-peptide complexes were obtained by dissolving 0.64 mg (peptide 12) or 0.7 mg (peptide 37) of lyophilized powder in 0.55 ml of the ubiquitin sample. The spectra were referenced indirectly with respect to external DSS (sodium 2,2-dimethyl-2-silapentane-5-sulfonate) with the Ξ coefficient equal to 0.101329118 for ^15^N nuclei (Wishart et al., 1995). All recorded spectra were processed by NMRPipe (Delaglio et al., 1995) and analyzed using Sparky software (Lee et al., 2015). The assignments of the amide peaks for the human ubiquitin were based on BMRB databank (BMRB 4493) and our previous studies. The ^15^N relaxation measurements were conducted with pulse sequences included in Agilent BioPack software (Agilent Inc. Palo Alto, USA) which was written on the base of previously published experiments (Farrow et al., 1994). The ^15^N R_1_ relaxation rates were obtained on the basis of eight experimental delays (10, 90, 170, 290, 410, 550, 690, and 850 ms). The ^15^N R_2_ relaxation rates were measured with the Carr–Purcell–Meiboom–Gill (CPMG) pulse train using 650 μs for refocusing time. The values were extracted from nine delays (10, 30, 50, 70, 90, 110, 130, 170, and 210 ms). The 3.0 s relaxation delay was employed in both experiments. ^15^N R_1_ and R_2_ relaxation rates were determined by non-linear least-squares fit using peak amplitudes to a single exponent curve. Errors were extracted from the covariance matrix. The ^1^H-^15^N NOE experiments were acquired with a recycling delay in 6 s. The ^1^H–^15^N NOE values were obtained as a ratio between peak heights in reference and saturated spectra. Errors were estimated on the base of signal-to-noise ratio in both spectra. Analysis of the evaluated relaxation data was performed according to Spectral Density Mapping (SDM) approach, which provides the spectral density function (J(ω)) at three independent frequencies –0, ω_N_ and 0.87ω_H_ (Farrow et al., 1995). Additional analysis was performed by determining the R_1_ and R_2_ product for selected residues exhibiting slow (μs–ms) dynamic motions (Kneller et al., 2002). The 2D ^1^H–^15^N HSQC spectrum collected for human ubiquitin is corresponding fully with the literature data. The sequence-specific assignments were performed on the base of BioMagnetic Resonance Databank (BRMB 4493) and previous 2D and 3D NMR experiments performed in our laboratory on ^13^C^15^N-uniformly labeled samples (Supplementary Figure 3). The recorded ^15^N relaxation data (R_1_, R_2_, and ^1^H-^15^N NOE) acquired at magnetic field 11.7T (Supplementary Figure 4) are in line with available the data previously recorded in another group (BRMB 6470). They confirmed that during the performed experiments, human ubiquitin occurred in properly folded state with a very stable 3D structure.

## Results

### Isolation of ubiquitin-specific peptide aptamers through deep DNA sequencing of combinatorial phage peptide library pools

**Figure 3:**
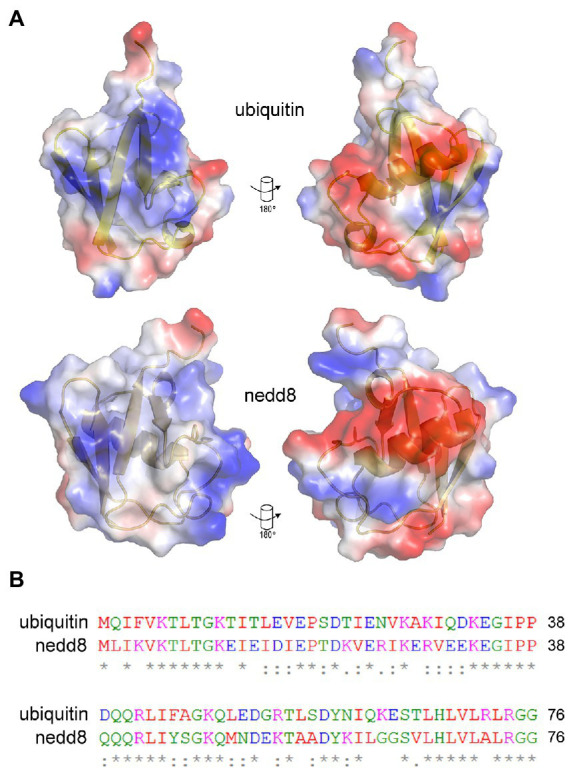
Similarities and differences between NEDD8 and ubiquitin proteins. **(A)** Charge density and structural similarities between ubiquitin and its ortholog NEDD8. **(B)** The amino acid sequence of NEDD8 and ubiquitin highlights a relatively high degree of divergence at the primary amino acid level. NEDD8 was used as a control to determine whether ubiquitin-specific peptides could be isolated using next-generation sequencing of peptide-phage library pools.

Recombinant human ubiquitin (with an N-terminal poly-Histidine tag) was immobilized on a nickel-coated plate in order to present a native or folded conformation in solution to the 12-mer combinatorial peptide-phage library. A control included the ubiquitin-like protein NEDD8 whose use would measure the specificity in the isolation of peptides to a structurally homologous protein but one with a different sequence and surface charge landscape (Figure 3). Three rounds of phage-peptide selection toward the two target proteins involved: (i) incubating the immobilized target proteins with the 12-mer peptide library for 1 h at 21°C; (ii) washing away non-specific or weakly bound peptides using two types of washes (either a 1 min (fast), low stringency wash or a 25 min (slow), high stringency wash to isolate peptides with a relatively high or low-off rate, respectively); (iii) eluting the specifically bound peptide-phage (unamplified phage-peptides); (iv) propagating the phage in bacteria (amplified phage-peptides), and (v) processing the amplified and unamplified phage DNA using RT-PCR DNA for next-generation DNA sequencing. Amplification of phages in bacteria after each round is necessary for multiplying the copy number of each clone to generate an enriched library that can be bio-panned again. Published results showed that amplification can enrich a subset of bacteriophage clones and thus identified a collapse of diversity after a single round of bacteriophage replication in bacteria (Matochko et al., 2012). As such, we focused our data analysis on next-generation ‘deep’ sequencing of peptide-phage processed directly from eluates (unamplified) to minimize amplified-based bias in peptide identification and/or motif discovery.

The bacteriophage DNA was extracted from each non-amplified or amplified M13 peptide-phage pool corresponding to each round and was then subjected to RT-PCR using DNA primers containing Roche 454 J adaptors as summarized in Figures 1A,B. Each PCR primer targeted a specific peptide phage pool and had a distinct internal triplet bar code that tags the DNA sequences to be extracted using Roche 454 next-generation sequencing datasets (Table 1; Figure 1B). A typical PCR product with an insert of the correct size of 429 bp is shown in Figure 1C (lane 2). An example of a PCR product with both insert (429 bp) and wild type (WT) phage (381 bp) is also shown (Figure 1C, lane 1). If WT-phage without insert contaminated a PCR reaction (as in Figure 1C, lane 1), then the upper band was excised for next-generation sequencing. Once all PCR products (from either Ubiquitin or NEDD8, rounds 1–3, low and high stringency washes, and included non-amplified and amplified peptide phage) were purified, then either 2.5 or 5 ng of gel purified DNA was pooled into one final sequencing pot containing 100 ng for next-generation sequencing (Supplementary Table 1). Unamplified phage isolated after elution became the major focus of analysis since these will not be biased by subsequent propagation in *E. coli*.

**Table 1:** A summary of the sequences of the primers used for amplifying each phage peptide pool.


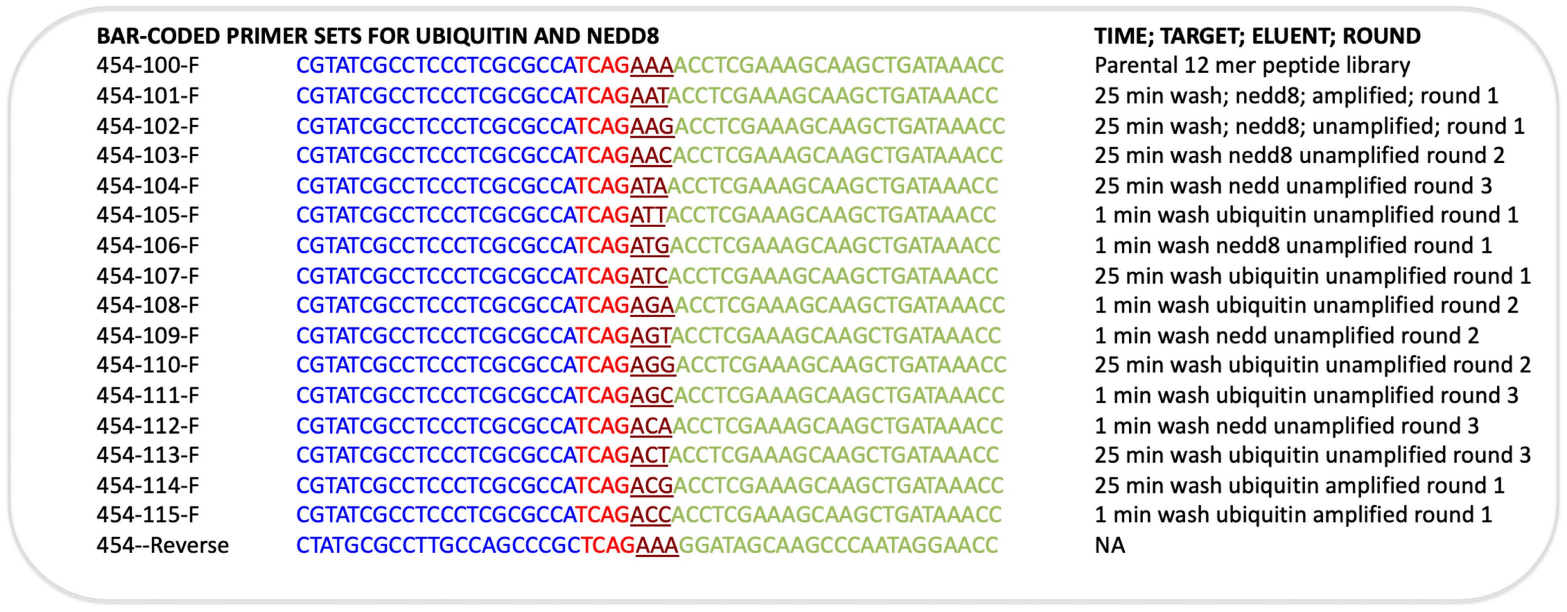

The bar-coded primers (3 base bar code in purple) are highlighted as a function of time of wash (in minutes), the target (nedd8 or ubiquitin), whether the phage pool was amplified or not amplified, and the round of bio-panning (1–3).

The DNA was processed by Next-Generation Sequencing methodologies according to 454 J protocols that can identify up to 70,000 sequences in the pooled amplicon series. Data were extracted from .fastq files where each sequence was associated with its sample identity (bar code is summarized in Table 1; Supplementary Table 2A–C summarize data for Ubiquitin and NEDD8, rounds 1–3, low and high stringency washes, and included non-amplified and amplified peptide phage, plus the parental library). In total, there were 40,451 total peptide sequences that were acquired from a theoretical total of approximately 70,000 sequences using the Roche 454 conjugation and amplification platform. The methodology is not confined to this sequencing platform as *Illumina* has also been used in phage display (Matochko et al., 2012). Deviations from the maximum using the Roche 454 platform relate to the presumed error in the calibration of the DNA concentrations to achieve approximately one oligonucleotide per bead coupled to the error in bead loss during the multiple washing steps. Of these 40,451 sequences, over 13,000 sequences were acquired from all rounds and washes using the Ubiquitin bar codes, and over 10,000 sequences were acquired from all rounds and washes using the NEDD8 bar codes (summarized in Supplementary Table 2C).

**Figure 4:**
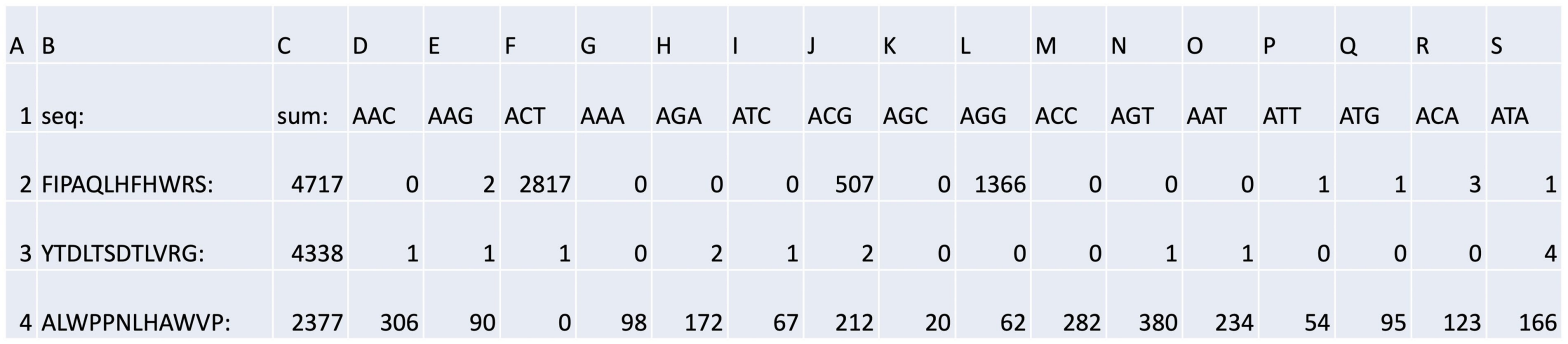
Bar code stratification of peptide counts. An example of the peptide sequences enriched against ubiquitin taken from Supplementary Table 2A. The data summarize the top sequences that were detected after next-generation sequencing was applied to the combined phage pools. The data are segregated based on (i) the rounds (1, 2, or 3); (ii) whether short or long washes were used; and (iii) the number of peptide sequences extracted from each bar-coded sequencing pool (The bar-coded data can also be observed in Supplementary Table 2A). The bar codes are in Table 1.

The output file containing the peptide sequences and the number of reads as a function of the bar codes was sorted as summarized in Supplementary Table 2A with a focused example in Figure 4. The peptide with the highest number of sequences identified (Figure 4, row 2, column C) was FIPAQLHFHWRS with 4,717 reads. Columns D through S contain the bar codes that match a particular round (1–3), target protein (ubiquitin or NEDD8), and stringency method (short or long washes). The bar codes are listed in Table 1. For example, in Figure 4, column F (ACT bar code), row 2 (FIPAQLHFHWRS sequence) tabulates 2,817 peptide reads from non-amplified phage. This represents the ubiquitin target, round 3, with a high stringency wash. Reads from the ubiquitin target, rounds 1 and 2, with a high stringency wash, using non-amplified phage, are in Figure 4, column I (ATC bar code) and column L (AGG bar code), respectively. Interestingly, using the ubiquitin target, with a high stringency wash, but using amplified phage from round 1, 507 FIPAQLHFHWRS peptide reads were acquired (Figure 4, row 2, column J, ACG bar code). However, as we did not sequence any ‘amplified’ phage after round 1, we do not know whether this sequence would have been enriched in subsequent rounds 2 and 3 using amplified phage. Most other columns in row 2 that focus on the FIPAQLHFHWRS peptide showed: 0 reads (such as column H); 1 read (such as column P); 2 reads (such as column E); or 3 reads (such as column R). These data highlight the relative specificity of FIPAQLHFHWRS peptide enrichment using ubiquitin and high stringency washes.

**Figure 5:**
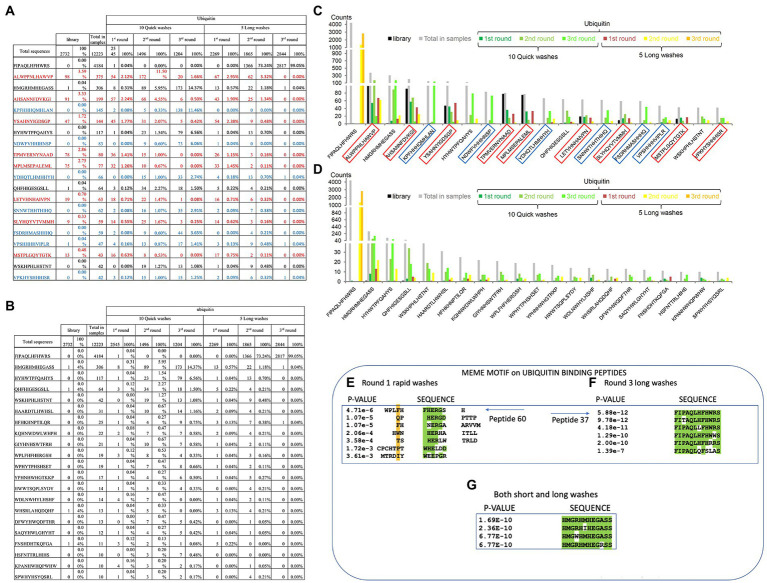
Specific peptide sequences enriched from Ubiquitin and NEDD8 peptide-library screens. **(A)** *Peptide sequences enriched against ubiquitin before removal of background peptides.* The data summarize the top sequences that were detected after next-generation sequencing was applied to the combined phage pools. The data are segregated based on **(I)** the rounds (1, 2, or 3); **(ii)** whether short or long washes were used; and **(iii)** the number of peptide sequences extracted from each bar-coded sequencing pool (The bar-coded data can also be observed in Supplementary Table 2A). Sequences in red reflect those peptides with a high representation in the parental library and are summarized as the total number of times the peptides were identified and as a percentage of the total sequences for that sequencing tag. For example, the sequence ALWPPNLHAWVP was identified 98 times out of 2,732 sequences with a parental library tag. The parental library bar code was AAA and this number of 98 counts of the sequence ALWPPNLHAWVP can be observed in Supplementary Table 2A. The sequences in red are defined as background due to their high abundance in the parental library and that they are not enriched during the selection process. The sequences in blue contain multiple histidine residues which were also removed to produce the final peptide lists. **(B)** *Peptide sequences enriched against ubiquitin after removal of background peptides.* The data summarize the top sequences that were detected after next-generation sequencing was applied to the combined phage pools and the background peptides (summarized in A) are removed. The data are segregated based on (i) the rounds (1, 2, or 3); (ii) whether short or long washes were used; and (iii) the number of peptide sequences extracted from each bar-coded sequencing pool. For example, from the long washes, the sequence FIPAQLHFHWRS was identified 0 times in round 1, 1,366 times in round 2, and 2,817 times in round 3. **(C,D)** The data from A and B are tabulated for visualization. The data are plotted as peptide count as a function of the indicated sequence. **(E–G)** The top 60 ubiquitin-selected peptides (from Supplementary Table 3) were evaluated using MEME (motif discovery; https://meme-suite.org/meme/tools/meme). As some examples: **(E)** Peptide 60 (motif collected from all peptides present in the Round 1 of Rapid washes) exhibited the core motif (W/F)HERG; **(F)** Peptide-37 (motif collected from all peptides present in Round 3 long washes) exhibited the core motif FIxAQLxFHWRS; and **(G)** Peptide 33 (from both short and long washes) exhibited the core motif HMGxHMHEGASS. In panels A-C, MEME derives a value of p based on parameters set in meme-suite (https://meme-suite.org/meme/tools/meme).

An example of what we would call a “non-specific peptide” sequence (ALWPPNLHAWVP, Figure 4, row 4, column C, with 2,377 total reads) reveals that there are relatively high levels of reads in every condition, except for Figure 4, row 4, column F (ACT bar code), with zero reads. This is the bar code representing ubiquitin, round 3, high stringency wash which had over 99% of the reads containing FIPAQLHFHWRS (see Figure 5); therefore, it is consistent that ALWPPNLHAWVP would show 0 reads on this frame.

We next processed these data to remove possible background peptide-binding activity (Figure 5). First, we removed peptides with three or more histidine residues since such peptides were defined to be background-binding peptides to the nickel plate used in the selection toward the his-tagged ubiquitin or his-tagged NEDD8 control target (Figure 5A, in blue). In addition, any peptides that were present more than one time in the parental library were also removed. For example, some unique peptides were found in higher copy numbers in the parental library itself such as ALWPPNLHAWVP which represented as much as 3.5% of the parental library (Figure 5A). Other peptides with high representation in the parental library include AHSANNFDVKGI, TPMVERNYNAAD, MPLMSEPALEML, or YSAHNYIGDSGP (Supplementary Tables 2A,B; Figure 5A). These peptides might be over-produced because they increase bacteriophage propagation in bacteria. As anticipated, some of these over-represented peptide sequences were specifically repressed in long washes and no significant enrichment observed using quick washes even if they remained abundant (Figure 5A). These same five peptides and other abundant peptides in the parental library are also observed in the NEDD8 screen (see Supplementary Table 2C).

Once such “background’ peptides were removed, the final list of peptides acquired is for Ubiquitin and NEDD8, respectively. A tabulation of the most enriched Ubiquitin-specific peptides is shown in Figure 5B. Notable was the peptide FIPAQLHFHWRS that showed enrichment in rounds 1, 2, and 3 of 0, 73, and 99%, respectively, of the total sequenced peptides from the long washes (Figure 5B). The data are quantified in graph form in Figures 5C,D. As control, the NEDD8 peptide enrichment (Supplementary Tables 2B vs. 2C) did not show significant similarity to the Ubiquitin peptide screen. One dominant peptide for NEDD8 binding was QHFHIGESGSLL with enrichment in each round seen only in the rapid washes (although binding was observed in the long washes; Supplementary Table 1). The peptide HYTHTHQYTYSM was also enriched in the long washes toward NEDD8. The ubiquitin-enriched peptide FIPAQLHFHWRS was observed in rounds 3 from NEDD8 selections but the penetrance was approximately two orders of magnitude lower than that observed when screening against Ubiquitin (Supplementary Table 1).

### Validation of ubiquitin-binding peptides

The peptide sequences enriched toward the Ubiquitin target can be stratified in several ways, apart from simple abundance. The first approach we developed was using MEME to identify motifs that emerge between the filtered, enriched peptides.^1^ The presence of motifs would suggest a binding specificity toward the target protein, as reported previously (Stevens et al., 2009; Mohtar et al., 2018). We focus here on two distinct groups of enriched peptides; (i) on peptides enriched toward ubiquitin in round-1_fast washes_not-amplified; (ii) on peptides enriched toward ubiquitin in both fast and slow washes_not-amplified; and (iii) on peptides enriched toward ubiquitin in round-3_long washes_not-amplified. These three states cover the three extremes in the peptide selection process; (i) lower affinities; (ii) intermediate affinities; and (iii) higher affinities, respectively. One peptide WPLFHFHERGSH that forms part of a cluster of peptides that derive a motif using MEME (Figure 5E) is relatively specific for enrichment in the fast washing stages (Figures 5B–D). Another peptide, FIPAQLHFHWRS, also forms a motif with additional peptides enriched in the screen with more stringent washes (Figure 5F). An example of another peptide that forms a top enriched peptide in either slow or fast washes toward Ubiquitin, HMGRHMHEGASS (peptide-33), also forms a consensus motif using MEME (Figure 5G).

### Validation of peptide aptamers as tools to manipulate an *in vitro* reconstituted ubiquitination system

We next evaluated whether these synthetic peptides (Supplementary Table 3) were able to impact on *in vitro* ubiquitination reactions. The initial reconstituted system was composed of ubiquitin, E1, UBCH5a (E2), E3 (MDM2), the p53 substrate, and an ATP regeneration system. Sixty of the most abundant synthetic peptides (Supplementary Table 3), were pooled into groups of 10 so that the molar ratio of any one peptide:Ubiquitin was 3:1. The reaction was balanced with a DMSO control and six reactions revealed two pools of peptide with significant inhibition of ubiquitination of p53 (data not shown).

Each 10-plex peptide pool was then divided into individual peptides so we could identify the bioactive peptide mediating inhibition of ubiquitination (representative data in Figure 2A). The most potent, reproducible inhibitors of ubiquitination were synthetic peptides 12 and 37 (Figure 2A, lanes 2, 4, and 7 vs. lane 1). Interestingly, peptide 37 has the sequence FIPAQLHFHWRS, which was the most highly significantly enriched in stringent screens (Figure 5B). Peptide 44 was inactive as ubiquitin inhibitors (Figure 2A, lane 5); this was an important negative control as it showed that a ubiquitin-binding peptide aptamer does not necessarily impact functionally on the ubiquitin transfer cascade.

In order to determine if the ubiquitination inhibition was linked to the E3 (MDM2) we evaluated these peptides in ubiquitination reactions using an unrelated ubiquitin ligase, CHIP (Figure 2B). Peptides 12 and 37 remained active in mediating inhibition of substrate ubiquitination (Figure 2B, lanes 4 and 5). This suggests that the peptides might be inhibiting at an earlier stage; (i) either E1 ubiquitin conjugation; (ii) ubiquitin transfer to E2; and/or (iii) release of Ubiquitin from E2 to substrate. As a control peptide 44 was inactive (Figure 2B, lane 3 vs. lane 2) as an inhibitor, indicating that synthetic peptides are not generally toxic to CHIP or the ubiquitination cascade in general. Future work will be carried out to dissect at what stage the peptides inhibit ubiquitination for example at the E2 discharge step.

In order to further validate the Ubiquitin-binding peptides, they were titrated into reactions above and below stoichiometric levels to Ubiquitin (Supplementary Figure 1A). The titration of both peptide-12 and pepide-37 reveals that near-complete inhibition of MDM2-dependent ubiquitination is observed at a 2:1 molar ratio of peptide:ubiquitin (Supplementary Figure 1A, lanes 3 and 6 vs. 2). However, at a ratio of peptide:ubiquitin of 0.2:1, there was no inhibition (Supplementary Figure 1A, lanes 4 and 7 vs. 2). These data suggest that inhibition by both peptides is not catalytic but involves direct sequestration of ubiquitin from the ubiquitin transfer reaction. If, for example, the peptides were active at a molar ratio of peptide:ubiquitin of 1:100, this might suggest that the peptide was inhibiting the E1-ubiquitin conjugated stage because E1 is present at 100-fold lower molar levels to Ubiquitin. The peptides were also tested against a more non-specific ubiquitination reaction involving RING domain only from MDM2 which lacks the p53 docking sites. Using this ubiquitination reaction, it is interesting to note that only peptide-37 inhibits the reaction (Supplementary Figure 1B, lanes 4 vs. 1). This could suggest that peptides 12 and 37 bind to Ubiquitin by different mechanisms and that the RING-only ubiquitination reaction does not involve a perhaps more complex protein–protein interaction as does CHIP or MDM2, both of which are inhibited by peptide-12 (Figure 2). However, these data can also be interpreted that peptide-37 has a higher affinity than peptide-12 which is why it inhibits RING-only ubiquitination.

### Orthogonal validation of ubiquitin-binding peptide-37 and peptide-12 using ELISA, NMR, and reverse phase chromatography

We next compared the relative binding of peptide-37 and peptide-12 to ubiquitin by ELISA using the same ‘sandwich’ format as the original screen which involved his-tagged ubiquitin added to the solid phase and peptide in the mobile phase. There is a weakness in the phage peptide screening concept that aims to acquire a useful tool. The phage peptide screen selects peptides binding when peptides are fused C-terminally to gIII protein of M13 and then the peptide has a free N-terminus after cleavage of the leader peptide (Figure 1). It is possible therefore that the peptide will only bind ubiquitin when it is fused to gIII because the gIII influences peptide conformation. This is not necessarily the case, as the bioactive peptides used in Ubiquitination assays had a, N-terminal tag and a C-terminal amide (Figure 2).

Nevertheless, we tested the binding of peptide-37 and peptide-12 in an ELISA format in which the peptides have a biotin-tag on the C-terminus of the peptide (Figure 2C). We realize that this C-terminal Biotin-tag does not mimic a gIII fusion, but it provides a useful tag in the event that is it active. The C-terminal biotin tag might mimic the peptide-phage in that the peptide is fused C-terminally to the tag of interest (Figure 1A). However, the final streptavidin-HRP:peptide complex might also deter binding by ELISA. Thus, it cannot be guaranteed that a peptide containing a tag on any orientation could even bind ubiquitin because of steric effects. Representative peptide-37 binding with C-terminal tag is shown in Figures 2C,D. In addition, as NEDD8 also has a similar structure to Ubiquitin, we tested peptide-37 binding by titration also in ELISA format and the data show that peptide-37 also binds NEDD8 but to a lower extent than Ubiquitin. For example, at lower peptide levels (12.5 ng) of peptide-37, corrected ECL units for Ubiquitin are 10,450 and for NEDD8 are 5,695 (Figure 2C). These data also highlight potential methodological problems in testing peptide binding by ELISA as an orthogonal assay. In this case, peptide-37 titration alone yields a relatively high background (Figure 2C, up to 47,545 avg. ECL units), while Ubiquitin alone also yields a relatively high background (up to 21,310 avg. ECL units). Peptide-12 did not bind by ELISA under these same conditions (data not shown). Similar results were obtained with a time course at fixed levels of peptide-37 (100 ng, Figure 2D). In this case, for example, by 60 min of binding, we detected 28,405 corrected ECL units for Ubiquitin and 30,173 corrected ECL units for NEDD8. Again, the background of Ubiquitin alone is relatively high at an average of 24,360 ECL Units (Figure 2D). Presumably, this indicates that there is some binding of the Ubiquitin to the Streptavidin peroxidase.

We finally attempted to employ other biophysical approaches to demonstrate whether peptides 37 or 12 bound to ubiquitin. We first attempted to define mode of binding of the peptides through analysis of peptic fragments using our previously published methods for measuring changes in ligand binding or mutational effects on MDM2 and CHIP E3 ubiquitin ligases using hydrogen-deuterium exchange mass spectrometry (Hernychova et al., 2013; Narayan et al., 2015). However, we found that ubiquitin was highly resistant to proteolysis by pepsin under these conditions (data not shown) and we were unable to acquire a comprehensive deuteration map of peptic ubiquitin peptides. As such, we sought to probe for global changes in overall deuteration of native ubiquitin in the absence and presence of each peptide. Deuterium exchange of ligand-free ubiquitin protein or in complex with peptide-12 or peptide-37 was initiated by dilution of ubiquitin into deuterated water. The molar ratios between ubiquitin and peptide-12 or peptide-37 were 1:2 and 1:5. Deuterium exchange was carried out for 1 min (Supplementary Figures 2A–F). The isotopic distribution of non-deuterated ubiquitin deconvoluted peak (calculated using *MagTran* (Zhang and Marshall, 1998) from charge states *z* = 7–12 and retention time 26.8–27.6 min) with an nominal average mass of 8564.85 Da (Supplementary Figure 2A). One minute of deuteration made a shift in global deuteration of ubiquitin resulted in an average mass of 8577.16 Da (Supplementary Figure 2B), together indicating an increasing in 12.31 deuterons. A reduction in the average mass of ubiquitin can be observed with increasing ratio of peptide-12:ubiquitin from 1:2 to 1:5 (Supplementary Figures 2C,D). Peptide-37 resulted in marginally elevated deuteration at peptide-37:ubiquitin ratio of 1:2, and an attenuated deuteration at a 1:5 ratio (Supplementary Figures 2E,F). These data together suggest that the peptides can bind to and have specific effects on the global deuteration of native ubiquitin. However, the data do not conclusively demonstrate a distinct and/or specific mode of action in the binding of peptide-12 or peptide-37 to Ubiquitin.

**Figure 6:**
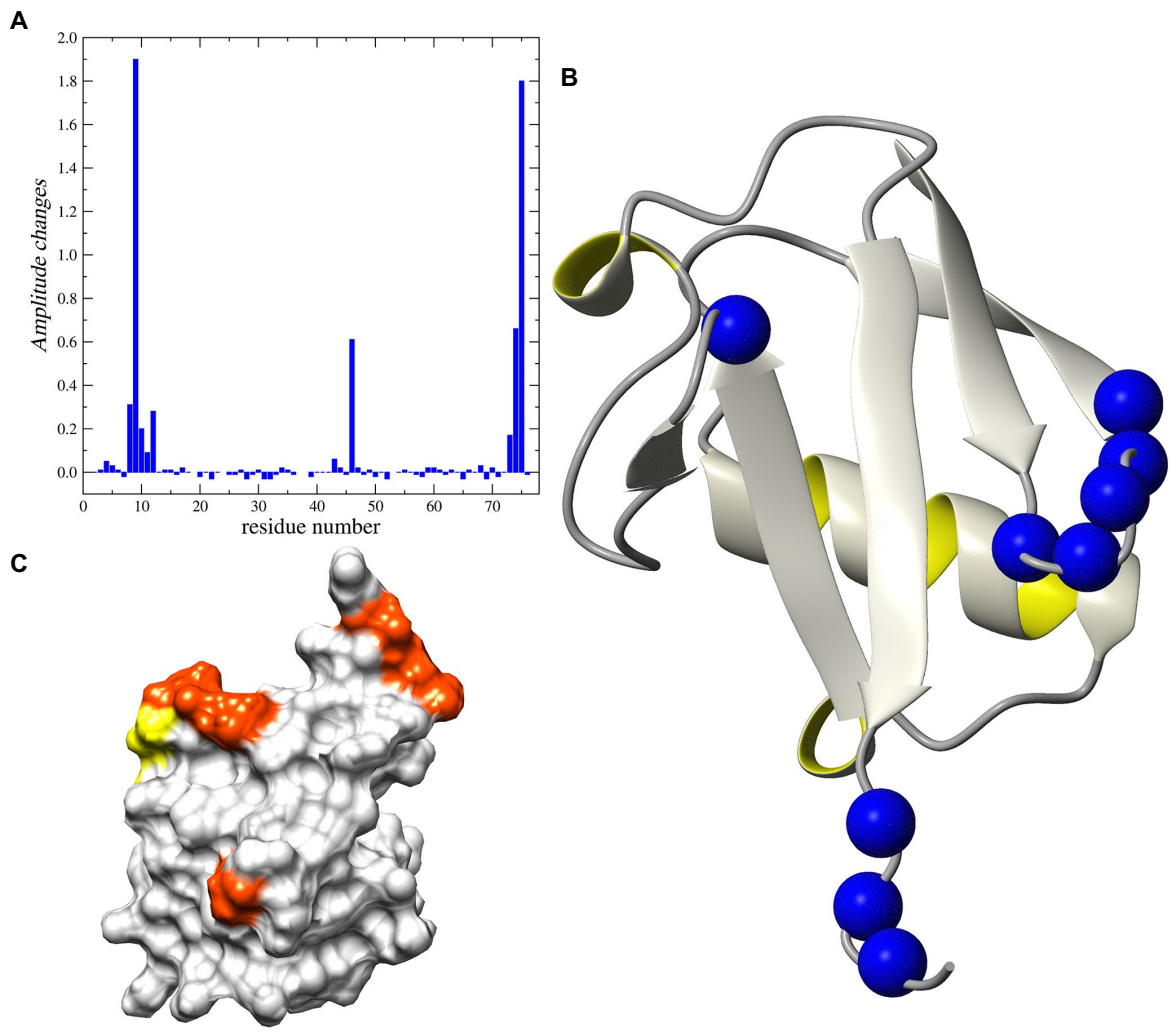
NMR analysis of Peptide-12 binding to ubiquitin. **(A)** Relative changes in intensity of ^1^H-^15^N HSQC cross-peaks induced by the addition of the peptide-12. **(B)** Ribbon representation of the 3D structure of human ubiquitin. The ^15^N nuclei in the residues corresponding to the increased peak heights are shown as blue balls. **(C)** The surface presentation of human ubiquitin. The positions of the residues demonstrating the increased peak intensity are highlighted in orange and yellow.

As such, NMR was used to determine whether we could detect binding of peptide-12 and peptide-37. The 2D ^1^H–^15^N HSQC spectrum collected for human ubiquitin corresponds fully with the literature and sequence-specific assignments were performed using the BioMagnetic Resonance Databank (BMRB 4493) and previous 2D and 3D NMR experiments performed on ^13^C^15^N-uniformly labeled samples (Supplementary Figure 3). The recorded ^15^N relaxation data (R_1_, R_2_, and ^1^H–^15^N NOE) acquired at magnetic field 11.7 T are in line with available data previously recorded (BMRB: 6470 BMRB 4493). The addition of the peptide-12 to the ubiquitin sample resulted in increased intensity of several cross peaks. Subsequently, characterizing the peptide-12 as selectively bound to ubiquitin along with substantial changes in peak heights detected for Thr9 and Gly75 residues (inserts on Supplementary Figure 3). A more in-depth analysis showed that these substantial changes include the β-turn located between first and second β-sheets (Leu8 – Thr12) and C-terminal fragment of the 3D ubiquitin structure (Arg72–Gly75; Figure 6). It is interesting that we did not detect any changes in the chemical shifts for amide groups of the ubiquitin (Supplementary Figure 3).

Analysis of the ^15^N relaxation data suggests an increase in the intensity of molecular dynamic processes at high frequency timescales (ns–ps) for residues located in C-terminal part of the protein, which is reflected in lower NOE values for residues starting from Arg72. The detected weak minimum for ^15^N R_2_ and NOE suggests an increasing amplitude of such a type of motions also in the fragment located between Thr9 – Thr12 (Supplementary Figure 4). The same residues exhibited changes in peak-height after peptide binding. On the other hand, there are two fragments of the protein that exhibited increased intensity of low-frequency motions (μs–ms) after binding peptide-12. Namely, the structural loop Ile23–Asn25 and the fragment around Ala46. The existence of the slow chemical exchange motions for the Ala46 was confirmed by the analysis of the R_1_ and R_2_ products (Supplementary Figure 5). That observation was further confirmed by analysis of the relaxation data using the SDM approach, revealing higher level of spectral density function observed for Ala46 at low and middle frequencies (*ω* = 0 and ω_N_; Supplementary Figure 6).

The addition of peptide-12 to ubiquitin resulted in a decrease in the intensity of molecular dynamic processes at low frequency time-frames (10^−3^ … 10^−6^ s) for the β-turn around Ala46 (Supplementary Figures 4–6). Another interesting observation involves the changes of the height of cross-peaks in ^1^H-^15^N HSQC spectra for the several residues grouped around Thr9 and Gly75 (Supplementary Figure 3). One interpretation of these data is that the increase in the peak intensities can be explained by binding the peptide-12 to selected residues in a fast-exchange regime, where the lifetime of the bound conformation is shorter than the lifetime of the unbounded (free) ubiquitin. The process of transition between the two states can cause the decrease in the exchange of amide protons with water for selected residues. The surface representation of the 3D structure of human ubiquitin reveals that all residues noted as possible binding sites for the peptides are located on one side of the protein (Figure 6). Our observations suggest that the peptide-12 interacts with ubiquitin specifically in the regions of loop Leu8–Thr12 and C-terminus (Leu73–Gly75). The peptide binding leads to the decrease of slow motion intensity for Ala46 in, most probably, an allosteric manner.

By contrast to peptide-12, the addition of peptide-37 to the ubiquitin sample was immediately detected in the NMR data. The signals in ^1^H–^15^N HSQC spectrum were characterized by increase of linewidth and decrease of signal-to-noise ratio (Supplementary Figure 7). The analysis of observed changes suggests the existence of a strong oligomerization process of human ubiquitin after incubation with the peptide-37. Extremely low signal-to-noise ratio excludes the possibility to collect any high-quality ^15^N relaxation data. Nevertheless, our estimations suggest that overall tumbling time (τ_m_) increased twice (at least), which corresponded with the effective molecular mass of ubiquitin oligomers around 25–30 kDa.

**Figure 7:**
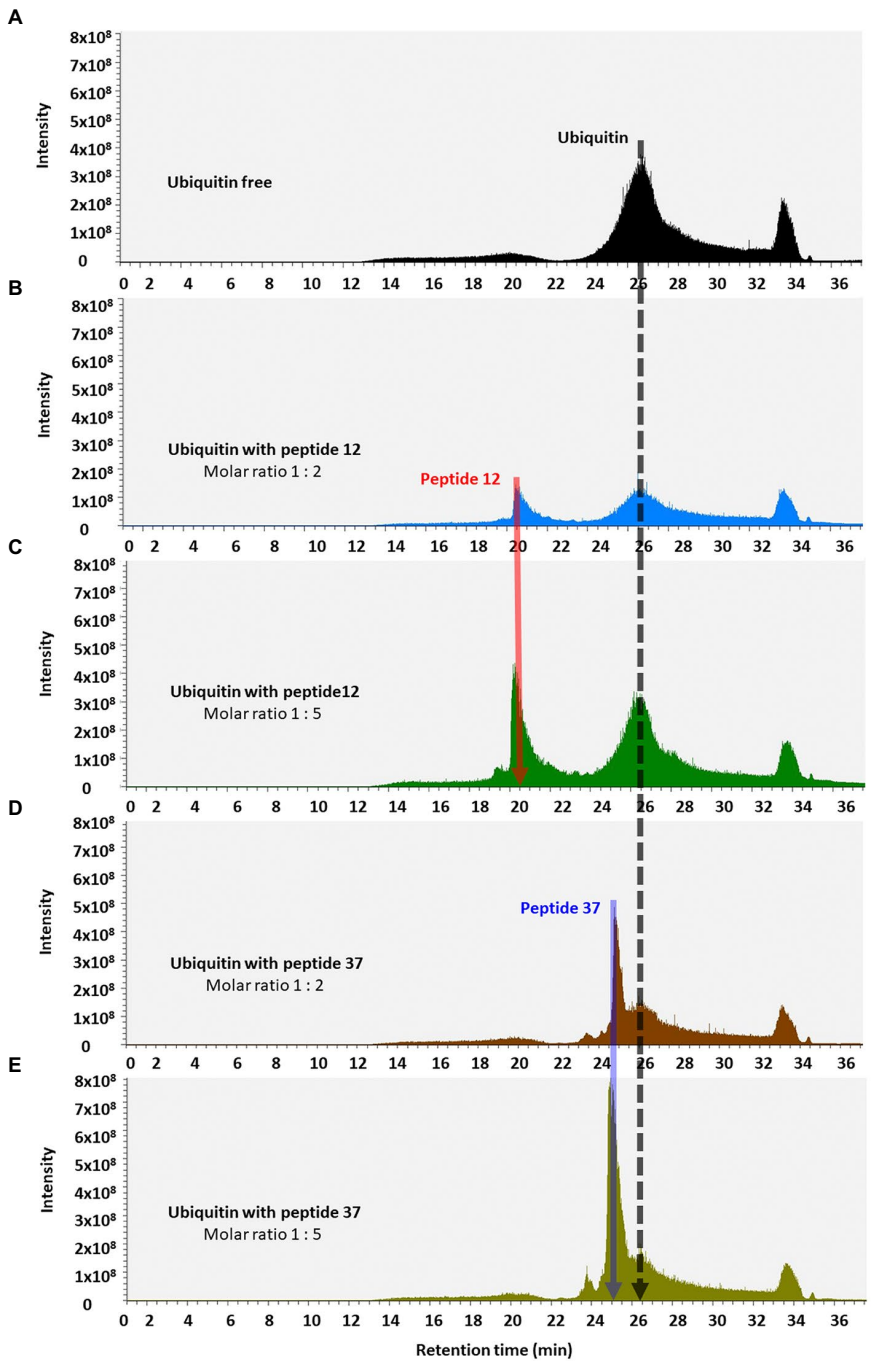
Total ion chromatograms of ubiquitin-peptide complexes. Ubiquitin was incubated alone **(A)** or with the indicated peptides **(B–E)**, diluted in D_2_O with 1% DMSO, and separated on a reverse phase C18 column according to the approach described in Methods. Total ion chromatogram was plotted as the relative abundance of the signal observed at a chosen m/z value as a function of RT. Material eluted at highlighted position and RT were confirmed by MS/MS analysis (Orbitrap Elite mass spectrometer) as the peptide-12 (red line, RT 20.25–22.00 min); the peptide-37 (blue line, RT 25.28–25.67 min), and ubiquitin (black line, RT 24.45–28.70 min).

**Figure 8:**
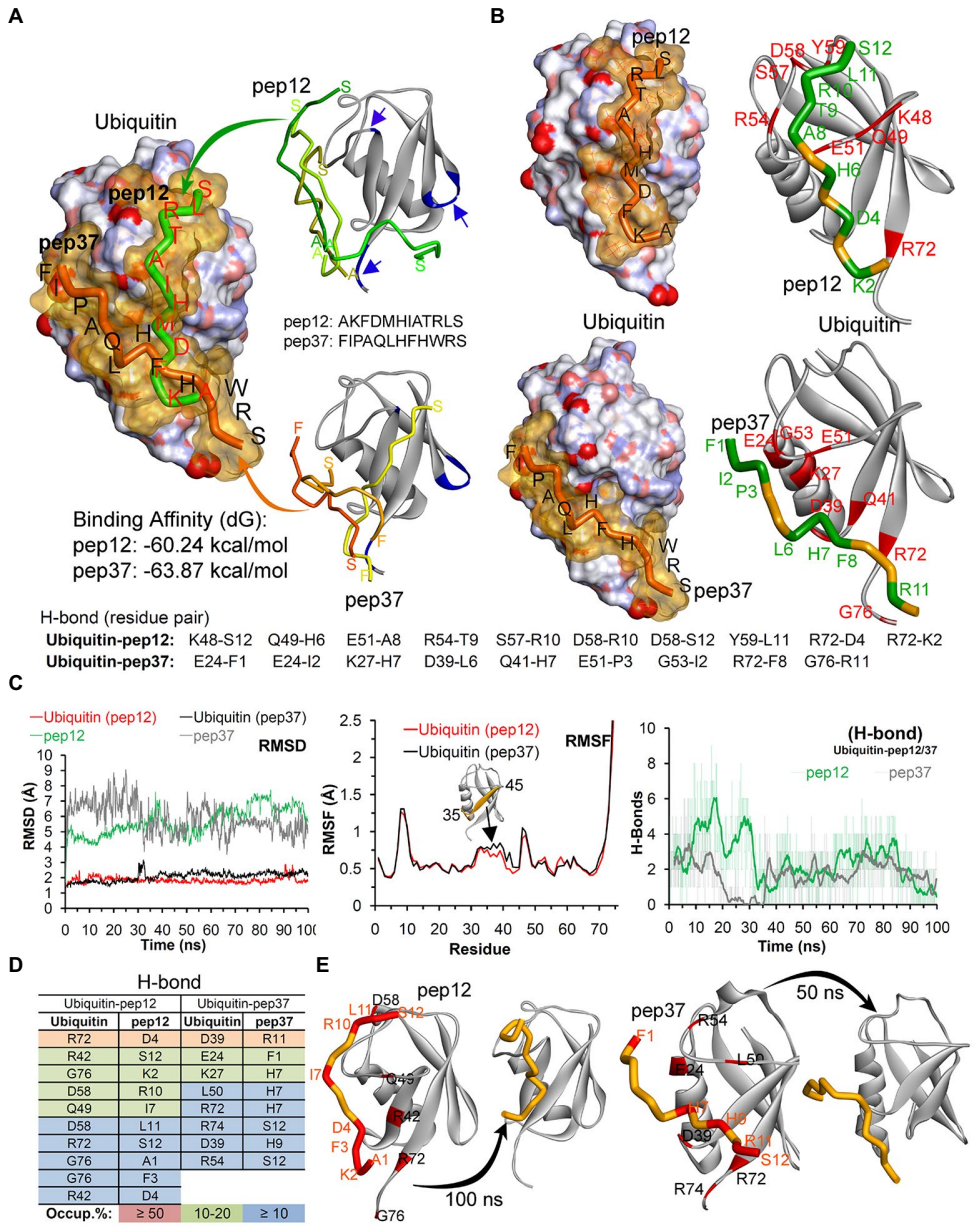
Model of peptide-12 and peptide-37 mediated inhibition of ubiquitination. **(A)** Peptides docked with ubiquitin having highest binding affinity are shown (blue arrows/region represent peptide-binding region traced in our NMR study). Although we were unable to determine whether the ubiquitin-binding peptides-12 or 37 bound to different or identical regions on ubiquitin, we analyzed potential conformations of peptide-12 and peptide-37 with ubiquitin by molecular dynamics simulation (MDS) and docking approaches. **(B)** The hydrogen bond (H-Bond) interaction map between amino acids of ubiquitin and peptides. **(C)** Structural changes and amino acid fluctuations identified for the Ubiquitin-peptide-12/peptide-37 complex using RMSD (root-mean-square deviation) and RMSF (root-mean-square fluctuation) calculations. **(D)** The stable intermolecular H-bonds between ubiquitin-peptide-12/peptide-37 over 100 ns of MDS, and residues pair with occupancy ≥ 5% (from 100 ns) are listed. **(E)** Structural dynamics of peptide-12 and peptide-37 observed during MDS. *In this figure, the ubiquitin crystal structure PDB id.: 1UBQ (60) in apo/inactive form was used, and ubiquitin is represented as atom charge surface/solid ribbon and pep12/pep37 are presented as tubes. Ubiquitin has a compact and rigid structure that ends in a flexible C-terminal tail. This rigid conformation allows only certain residues to have limited flexibility to permit a large diversity of protein–protein interactions (Komander and Rape, 2012; Ernst et al., 2013). It contains a hydrophobic core formed divided in three areas: the first one around Ile44, Leu8, Val70 and His68 is involved in most of the interactions with ubiquitin-binding proteins (UBP) and the proteasome (Sloper-Mould et al., 2001; Dikic et al., 2009); the second patch, around the residues Ile36, Leu71 and Leu73 is important for recognition by some HECT E3 ligases (Kamadurai et al., 2009). Finally, the area around Gln2, Phe4 and Thr14 interacts with certain proteins including some DUBs (Hu et al., 2002).

We finally used reverse-phase HPLC (C18) as a methodology to determine whether Peptide-37 induces an aggregation-like property to ubiquitin as suggested by NMR. Reverse-phase HPLC has been used previously to identify conformational changes in a target protein. For example, insulin (mass of approximately 5,300 Da) from different species can be separated by reverse-phase HPLC when differing by only a serine and threonine residue (Rivier and McClintock, 1983). Interleukin mutant retention times on reverse phase-HPLC have been correlated to structural changes (Kunitani et al., 1986). The molar ratios between ubiquitin and peptide-12 or peptide-37 were 1:2 and 1:5. The total ion chromatograms (TIC) of ubiquitin-peptide complexes are shown in Figures 7A–E. TIC highlighted that three of the most abundant ion signals corresponded to the peptides (peptide-12 had a retention time (RT) of 20.47–21.51 min and peptide-37 had a RT of 25.48–28.86 min). The other two ion signals represent co-elution of ubiquitin and the respective peptides (RT 25.48–28.86 min and 33.34–34.34 min; mass spectral data not shown). The retention time of ubiquitin either alone or in complex with both peptides remains the same (RT 24.45–28.70 min). However, the absolute peak intensities of the eluted ubiquitin are different. In particular, the intensity of the ubiquitin signal is compressed when it is in complex with peptide-37 at a 1:2 or 1:5 molar ratio of protein: peptide (Figures 7D,E vs. Figure 7A), which is characteristic of protein loss during chromatography, perhaps due to ‘aggregation’. The impact of peptide-37 on apparent aggregation or oligomerization of ubiquitin during chromatography is in agreement with NMR data. A model of peptide-12 and peptide-37 docking using molecular dynamics simulation (MDS), hydrogen bond (H-Bond) interaction map, and structural changes and amino acid fluctuations identified for the Ubiquitin-peptide-12/peptide-37 complex using RMSD (root-mean-square deviation) and RMSF (root-mean-square fluctuation) calculations are in Figure 8, which suggests different modes of binding.

## Discussion

Discovering protein–protein interactions is an important goal in life sciences (Morelli and Hupp, 2012). The number of interacting proteins for any given eukaryotic protein can be relatively large. Classic examples include the ATM/ATR kinases which are reported to have over 700 substrates (Matsuoka et al., 2007). It is becoming apparent that one of the main drivers of such a diversity of protein–protein interactions are linear motifs that form weak, but specific protein–protein interactions important in cell signaling. Linear motifs form vast numbers of combinatorial protein–protein interaction interfaces that offer a rich source of signaling diversity (Tompa et al., 2014). Thus, advances in approaches for discovering functional linear peptide motifs is important for the protein science field.

Peptide combinatorial libraries could provide a powerful platform for high throughout linear peptide motif discovery. The power of massively diverse combinatorial peptide phage display lies in its ability for an efficient and rapid identification of peptide ligands from a phage population displaying millions of diverse surface peptides. In addition, the reliance on *in vitro* screening is also an advantage in that the bait conformation, buffers, and selection conditions can be applied selectively. *In vitro* selection previously conditions identified MDM2 protein interactors; pre-incubating MDM2 with its ligands including zinc or RNA can give rise to the isolation of distinct linear peptide motifs from phage-peptide populations (Shimizu et al., 2002; Burch et al., 2004). However, a major disadvantage of the phage-peptide pool has been the relatively low throughout in peptide identification by DNA sequencing. Typically, only dozens of peptides are identified from sequencing using standard Sanger sequencing platforms (Murray et al., 2007). Next-generation sequencing methods could create a platform in which the diversity of the peptide-phage library can be fully exploited through the sequencing of thousands or millions of peptide inserts. In this report, we set up a next-generation sequencing platform on a peptide-phage library screen that can sequence using Roche454J platforms up to 70,000 inserts from one reaction (Figure 1). Our data demonstrate that using next-generation DNA sequencing, peptide library diversity can be defined, background (non-specific) peptides can be identified, and enrichment can be quantified isolating peptides with a high degree of specificity. Illumina sequencing of peptide-phage populations was used previously to our study (Matochko et al., 2012), thus providing greater sequence depth to the platform we use in this report.

E3 ubiquitin ligases can select and ubiquitinate client proteins by complex mechanisms that have not been fully dissected. For example, allostery in the CHIP E3 ubiquitin ligase was revealed by biophysical characterization including hydrogen-deuterium exchange mass spectrometry that demonstrated conformational-inhibition-signals extend from the TPR-domain to the U-box (Narayan et al., 2015). The MDM2 E3 ubiquitin ligase exhibits complex allosteric effects that exploit multiple protein–protein docking sites on the p53 substrate and the co-factor, E2 (Wallace et al., 2006; Wawrzynow et al., 2009; Fraser et al., 2015). The stoichiometry and dynamics of how the E1–E2–E3 conjugation system operates is not well understood; for example, in the case of MDM2 it is not clear if substrate docking involves the formation of a dimeric MDM2 structure (Robson et al., 2012). How ubiquitin itself is detected, orientated, and conjugated is not completely defined and it is likely to be highly combinatorial (Ernst et al., 2013). Conjugation of mono-ubiquitin to a transcription factor can present a face of ubiquitin that can interact with DNA (Landre et al., 2017). As such, we applied our next-generation peptide-screening platform to human ubiquitin, a core substrate in the ubiquitin conjugation reaction, to determine if peptides can be isolated that bind to and impact on ubiquitination. We identified using deep-sequencing a small number of specific peptide aptamers that were useful as tools to begin to explore steric constraints in the ubiquitin conjugation reaction. For example, two peptides we identified were studied (named 12 and 37). Analysis of these two peptides revealed that, although they both inhibit ubiquitination at similar molar ratios (Supplementary Figure 1A), they differ in these orthogonal assays; (i) ELISA’s show difference in binding to ubiquitin; (ii) RING domain-only ubiquitination is specific for peptide 37; (iii) HPLC analysis of peptide-ubiquitin complexes reveal a difference in ubiquitin peak intensity; and (iv) NMR suggests different binding modes with peptide-37 stimulating apparent aggregation. Further biophysical studies would be required to determine if the peptide-12 and peptide-37 bind by different mechanisms to Ubiquitin or whether these differences reflect changes in affinity to the target. Nevertheless, these orthogonal assays are presented to provide readers, of this methodological study, some ideas on orthogonal biochemical assays that can be used to test synthetic peptides, derived from phage display, when the peptides are not fused to gIII of M13 phage.

Previous research has highlighted the conformational dynamism of the ubiquitin conjugation cascade and the importance of weak ubiquitin interfaces (Haririnia et al., 2008). The E2 ubiquitin transfer proteins use a reactive cysteine that forms a covalent adduct with ubiquitin through a thioester bond. The stability of the E2-ubiquitin conjugate is maintained by conserved asparagine that stabilizes the oxyanion transition state (Wenzel et al., 2011). A low-affinity protein–protein interaction between ubiquitin and E2 maintains steric occlusion of the active site to permit nucleophile attack of the thioester bond and orientates the incoming ubiquitin to catalyze ubiquitin chain linkage (Saha et al., 2011). A small chemical molecule (CC0651) was identified that binds the E2 conjugation protein CDC42 and this molecule blocks ubiquitination transfer by virtue of trapping a weak interaction between ubiquitin and the E2 (CDC42) ubiquitin-binding site (Huang et al., 2014). The data suggested that stabilization of other weak interactions between ubiquitin and ubiquitin conjugation enzymes by small molecules could drive drug-discovery in the UPS system. Indeed, it is interesting that one of the peptides identified in our study (peptide-37) also attenuated ubiquitin-E2 hydrolysis (data not shown) suggesting that this thioester conjugate stability can be targeted by ligands that bind E2 or ubiquitin. We have also identified natural product extracts that we also show can mimic the peptides and impact on the E2-ubiquitin discharge (data not shown) and further suggests that the E2-ubiquitin complex can be manipulated by several types of bio-chemical interventions.

A final utility of the deep sequencing of linear peptide libraries is the opportunity to produce a consensus site that drives discovery of authentic and novel protein–protein interactions. The use of motif search engines can result in the identification of novel protein–protein interfaces that can be validated using emerging robust platforms for *in situ* measurement of endogenous protein–protein interactions (Dinkel et al., 2014; Gibson et al., 2015). Using the consensus site of peptide-37 (Figure 5), we can similarly identify several proteins in the human proteome with this motif (data not shown) and some of these targets are currently being validated as novel ubiquitin-binding adaptor proteins (unpublished data). Thus, deep DNA sequencing of combinatorial peptide libraries can therefore be used to (i) identify peptide aptamers to a target protein for use as tools to manipulate and study protein–protein interaction dynamics and (ii) discover new protein–protein interfaces of potential biological relevance.

## Conclusion

This study provides a methodological blueprint for using next-generation sequencing of peptide-phage library pools screened against a target protein. In addition, orthogonal assays that are presented to characterize enriched peptides include (i) synthesis of peptides with an N-terminal or C-terminal Biotin tag to test activity in binding to target protein by ELISA; (ii) testing peptide activity in an enzymatic assay; (iii) measuring the effects of peptides on target protein binding by NMR; (iv) examination of the effects of peptides on target protein deuteration; and (v) possible mode of binding of peptides onto target using MDS.

## Data availability statement

The original contributions presented in the study are included in the article/Supplementary material, further inquiries can be directed to the corresponding author.

## Author contributions

ML: investigation and validation. FL: data curation, investigation, and writing—original draft. MG-M: investigation and data curation. A-SH, LH, and AK: methodology. LW: writing—review and editing. PM, BV, RK, IZ, and PJ: resources. SR-M: conceptualization and writing—review and editing. KB: supervision. TH: funding acquisition, supervision, and conceptualization. UK: software, visualization, and supervision. ZT: investigation. All authors contributed to the article and approved the submitted version.

## Funding

This work was supported by the European Regional Development Fund—Project ENOCH (No. CZ.02.1.01/0.0/0.0/16_019/0000868), by the Ministry of Health Development of Research Organization, MH CZ—DRO (MMCI, 00209805). BV, PM, LH, and RK were supported by Czech Science Foundation (No: 19-03796S). MG-M was funded by Scottish Power. A-SH was funded by Cancer Research UK (C483/A10706). LW was supported by BBSRC (BB/K011278/1). UK was supported by grant 2020/36/C/NZ2/00108 from The National Science Centre, Poland (Narodowe Centrum Nauki, Krakow, Poland). The International Centre for Cancer Vaccine Science (ICCVS) project is carried out at the University of Gdańsk within the International Research Agendas programme of the Foundation for Polish Science co-financed by the European Union under the European Regional Development Fund (FL, ML, and UK).

## Conflict of interest

The authors declare that the research was conducted in the absence of any commercial or financial relationships that could be construed as a potential conflict of interest.

## Publisher’s note

All claims expressed in this article are solely those of the authors and do not necessarily represent those of their affiliated organizations, or those of the publisher, the editors and the reviewers. Any product that may be evaluated in this article, or claim that may be made by its manufacturer, is not guaranteed or endorsed by the publisher.

## Footnotes

^1^ https://meme-suite.org/meme/tools/meme
