# Supplementary material for: Next-generation sequencing of a combinatorial peptide phage library screened against ubiquitin identifies peptide aptamers that can inhibit the in vitro ubiquitin transfer cascade
Source: Front Microbiol. 2022 Dec 2;13:875556. doi: 10.3389/fmicb.2022.875556 (PMC9755681; doi:10.3389/fmicb.2022.875556)
Supplement: Supplementary file 6 [file Data_Sheet_1.docx]

**Supplementary Figures**

**Supplementary Figure 1**

**Supplementary Figure 2**


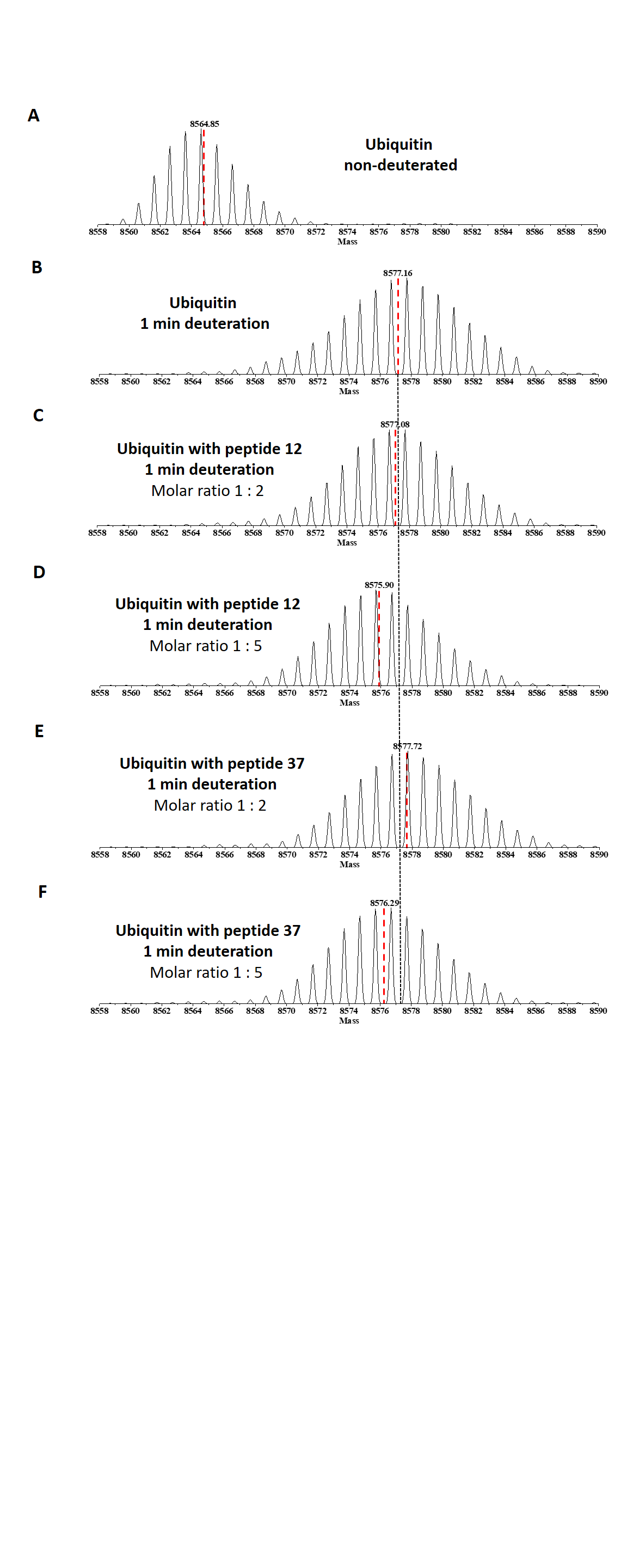


**Supplementary Figure 3**
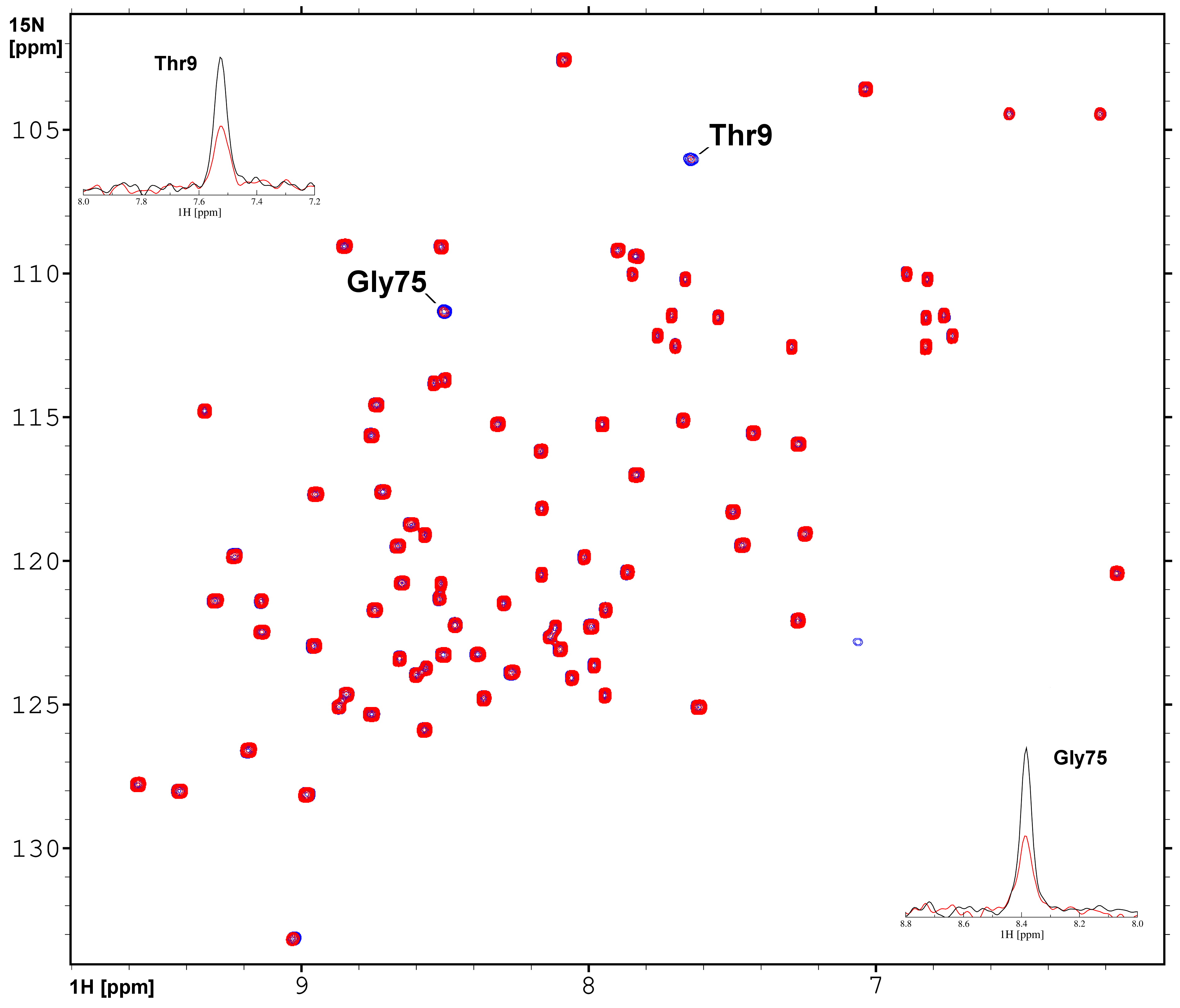


**Supplementary Figure 4**
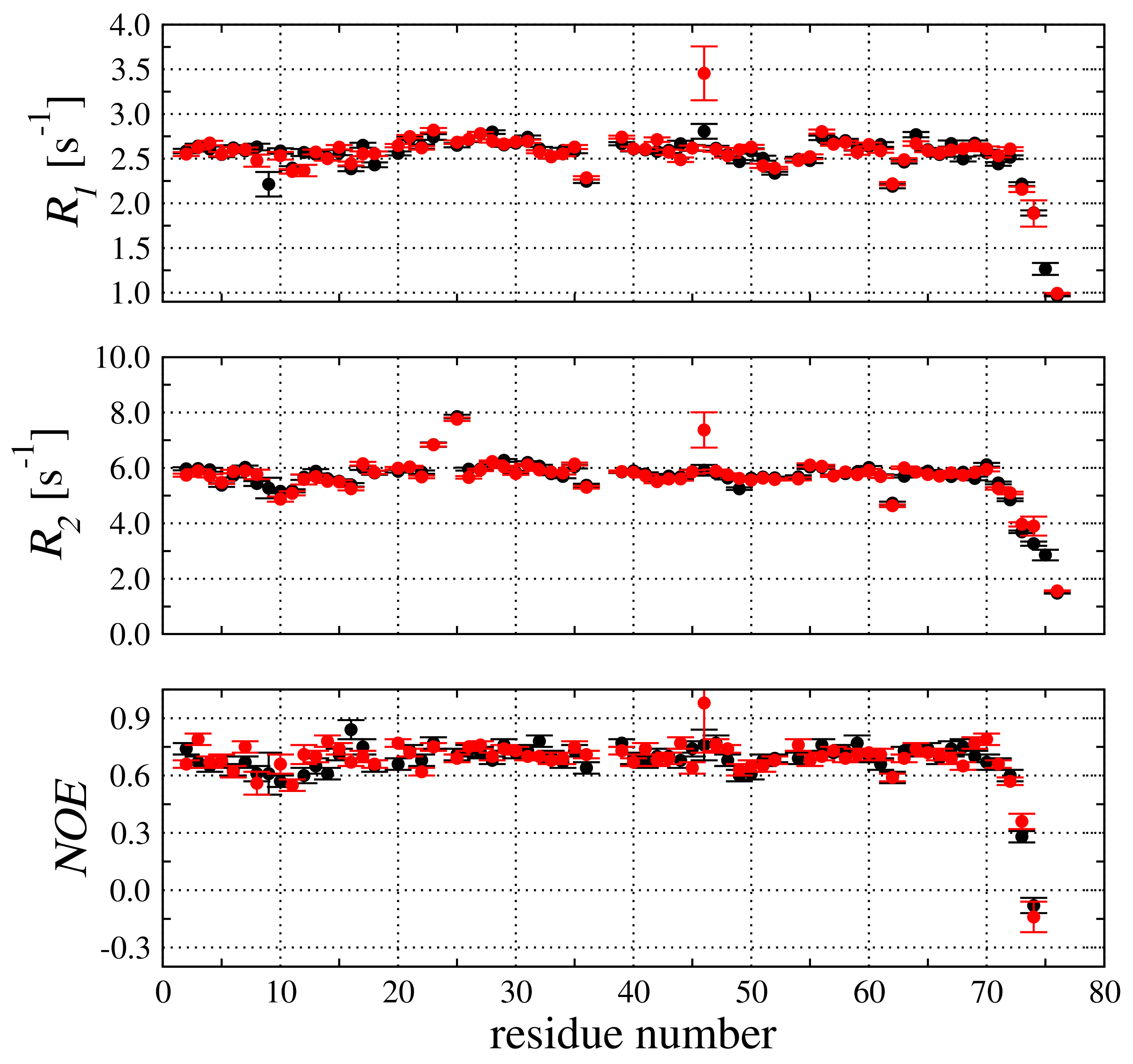


**Supplementary Figure 5**


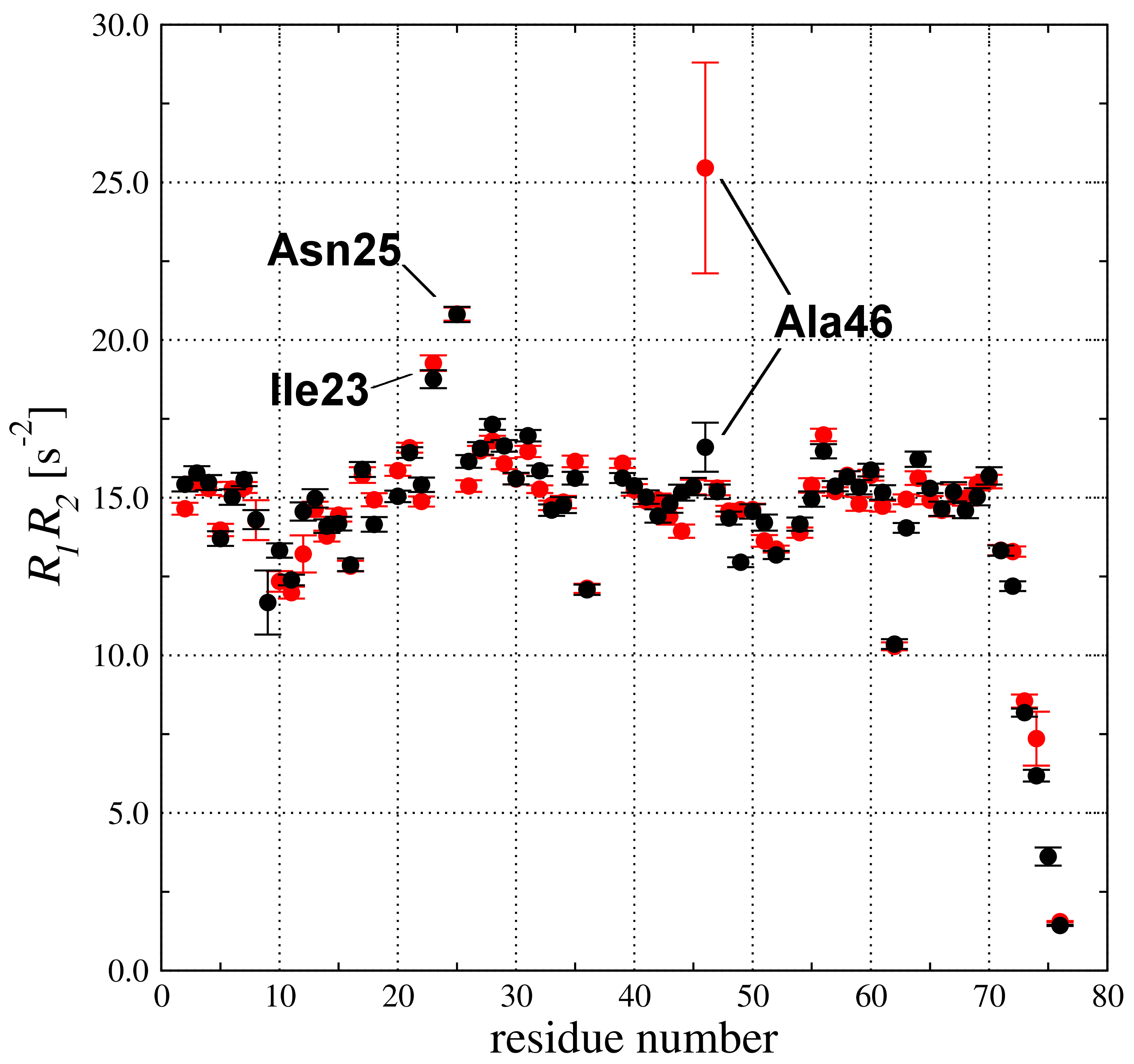


**Supplementary Figure 6**
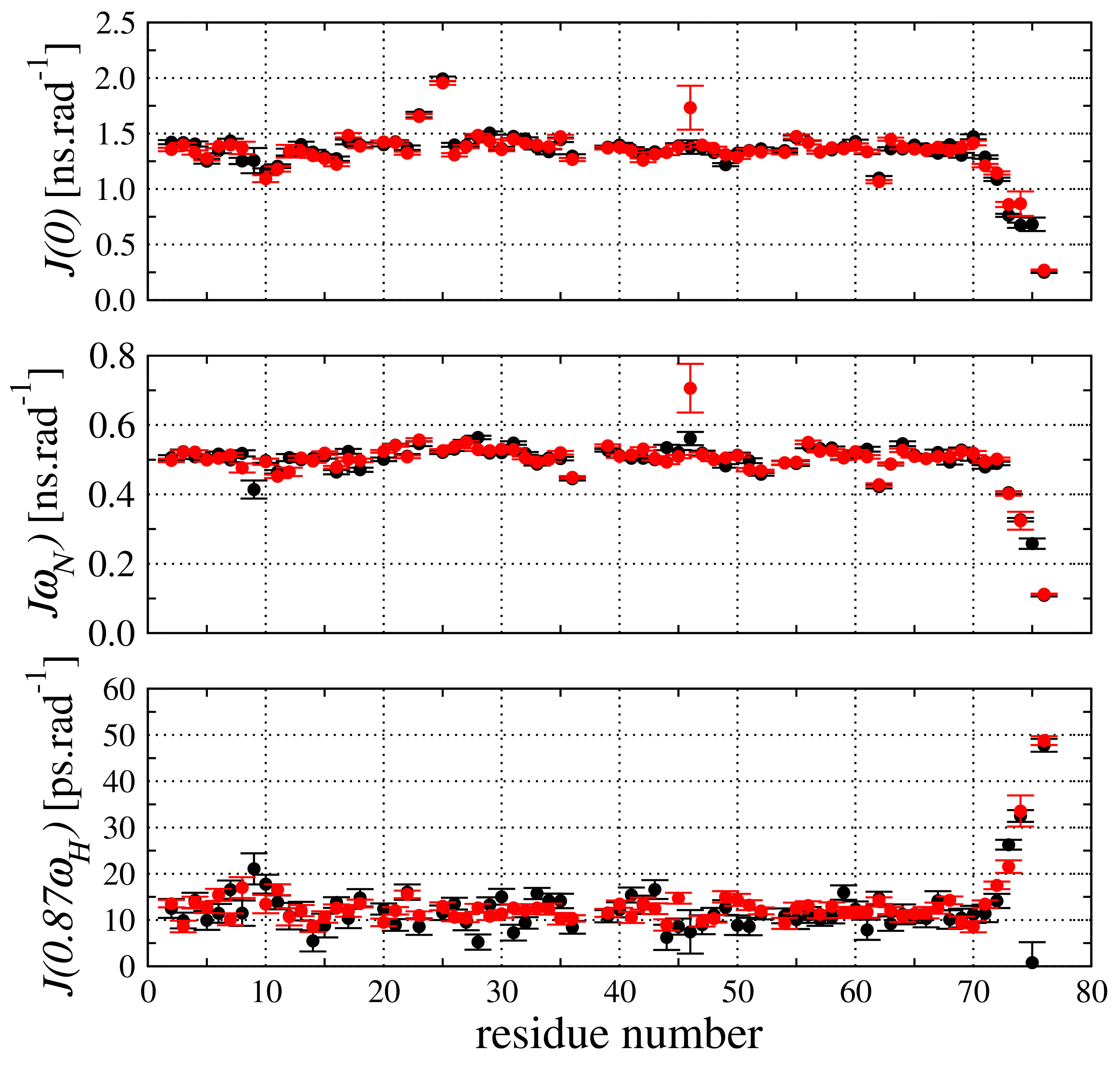


**Supplementary Figure 7**


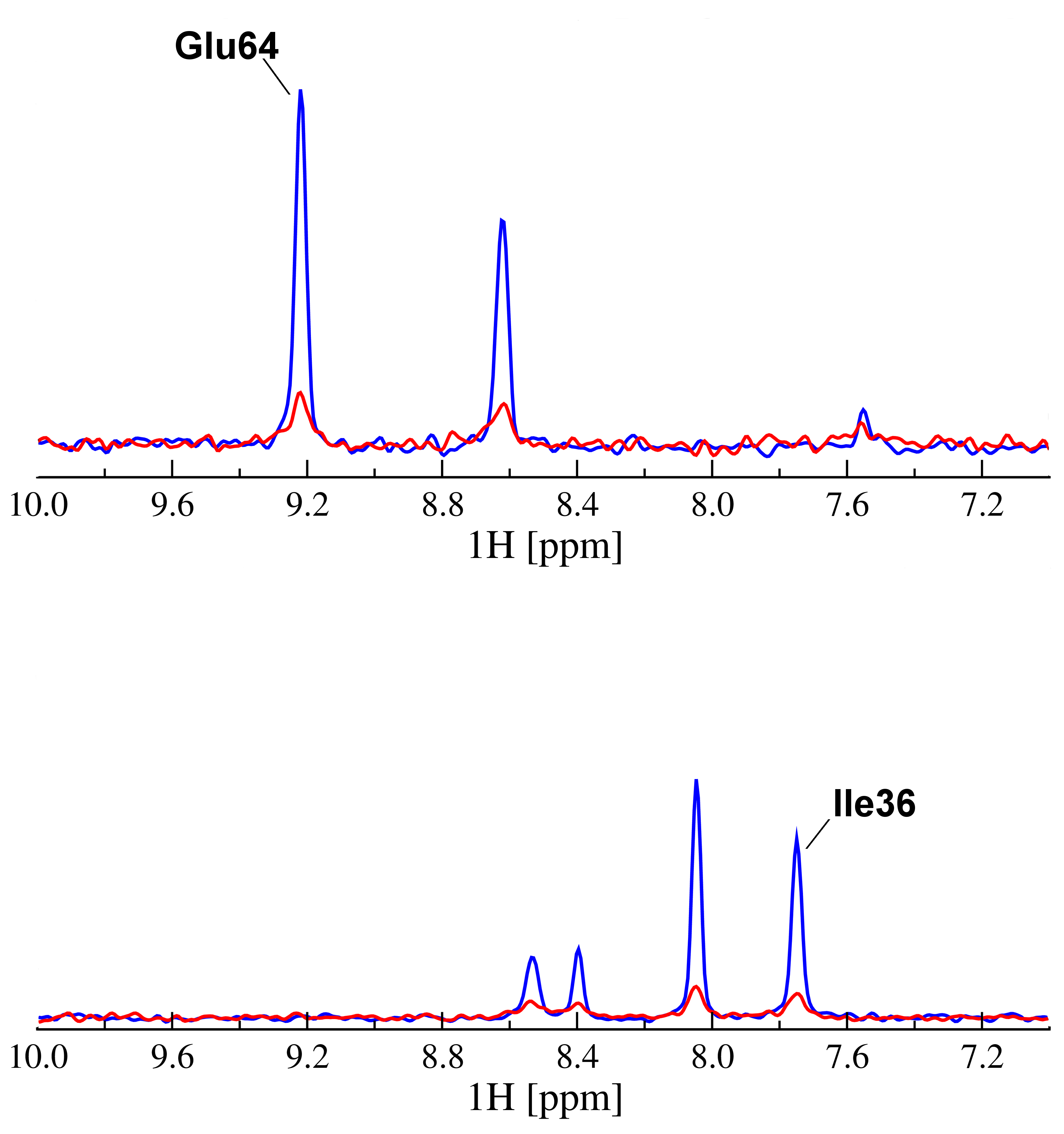


**Supplementary Figures Legends**

**Supplementary Figure 1. Evaluation of peptides with bioactivity in orthogonal ubiquitination assays.** (A). Ubiquitin assays driven by the E3 ubiquitin ligase MDM2 were assembled with changes in the molar ratio of peptide:Ubiquitin. The active peptides 12 and 37 were evaluated. Reactions products were immunoblotted with antibodies to the substrate p53. (B). Ubiquitin assays driven by the RING domain only from MDM2 were assembled to evaluate the effects of peptides 12 and 37 in ubiquitination. Reaction products were immunoblotted with antibodies to the substrate p53.

**Supplementary Figure 2.** Global analysis of ubiquitin after peptide interactions. Deconvoluted ESI spectra of ubiquitin charged ions (z = 7-12 in the extracted ion chromatograms with retention time 26.8-27.6 min. (A). The isotopic distribution of undeuterated and ligand-free ubiquitin with average mass 8564.85 and (B). One minute deuterated ubiquitin with average mass 8577.16 (12.31 hydrogens were exchanged to deuterons). (C-F). The isotopic distributions of deuterated ubiquitin assigned with average masses were measured with the indicated molar ratios of ubiquitin:peptide of 1:2 and 1:5. Black dashed line shows differences among deuteration states B-F. The data are plotted as relative abundance as a function of m/z ratios.

**Supplementary Figure 3.** Overlay the ^1^H-^15^N HSQC spectra recorded for human ubiquitin before (red) and after addition of peptide 1 with the molar ratio of 1:2 (blue). The cross-peaks for the Thr9 and Gly75 demonstrating significant changes in the peak amplitudes are marked. The corresponding 1D traces along ^1^H axis for Thr9 and Gly75 are presented as inserts.

**Supplementary Figure 4.** ^15^N relaxation data (R_1_, R_2_, ^1^H-^15^N NOE) collected for human ubiquitin before (red) and after saturation with the peptide 1 with the molar ratio 1:2 (black).

**Supplementary Figure 5.** The ^15^N relaxation data displayed as R_1,_R_2_ for human ubiquitin before (red) and after addition of the peptide 1 (black). The residue Ala46 exhibited notable amount of slow mobility which vanished after the addition of peptide 1. The residues Ile23 and Asn25 are highlighted as exhibiting higher mobility for the N-terminal part of α-helix.

**Supplementary Figure 6.** Analysis of ^15^N relaxation data with the Spectral Density Mapping (SDM) approach. The spectral density function for human ubiquitin before (red) and after saturation with the peptide 1 (black) calculated for the following frequencies: 0 (J(0)), ω_N_ (J(ω_N_)), and 0.87ω_H_ (J(0.87ω_H_)).

**Supplementary Figure 7.** The traces along ^1^H dimension corresponding to ^15^N resonance frequencies for Glu64 (top) and Ile36 (bottom) extracted from the ^1^H-^15^N HSQC spectra acquired for ubiquitin saturated with peptide 1 (blue) and peptide 2 (red). Although Peptide-12 induced increased intensity of the cross peaks suggesting that the detected interactions are characterized by a fast exchange regime (meaning that the exchange rate (k_ex_) between bounded and free states is much faster than NMR timescale), peptide-37 induced protein oligomerization making the relaxation data impossible to acquire. Nevertheless, these data suggest that Peptide-12 and Peptide-37 interact with Ubiquitin by distinct mode of binding.

**Supplementary Tables Legends**

**Supplementary Table 1**. An example of the DNA concentration (in ng/ul) of each library pool using all 16 forward bar-coded primers (from Table 1, 00 through 15) and four additional bar coded primers (TAG, TAA, TAC, TAT) to a control protein that is not discussed in this study. Primers 00 through 15 were focused on the ubiquitin and nedd8 targets and primers 16-19 were applied to a non-specific protein as a control (data not shown). The table highlights whether the pool was amplified (a) or non-amplified (u) prior to PCR amplification for gel purification. The round label (1-3, or 4 for the one control using primer F19), whether the wash was low or high stringency, and the volume required to achieve either 5 ng or 2.1 ng of input DNA are also listed in each column. Using all 20 pooled samples, a total of 19.99 ul of sample was collected, at a concentration of 5 ng/ul, with a total amount of DNA equal to 100 ng. This material was processed for next-generation DNA sequencing.

**Supplementary Tables 2A, 2B, and 2C.**(A). Converted sequences. Raw DNA sequences processed by Next-Generation Sequencing methodologies were extracted from fastq files where each sequence was associated with its sample identity (yellow columns; bar codes are from Table 1, and as indicated in the excel Table in row 1). Listed are the number of peptides sequenced as a function of peptide sequence and bar code from (i) the parental library; and (ii) from rounds 1-3 of screening including low and high stringency washes and included non-amplified and amplified peptide phage. For example, the column with the bar code AAA represents the parental library and there were 0 peptides with the sequence of peptide-37 (FIPAQLHFHWRS). The column with the bar code ACT represented 3^rd^ round long washes with Ubiquitin and there were 2817 peptides with the sequence of peptide-37. Column A lists the sequence and is sorted by the number of times a peptide is identified, with FIPAQLHFHWRS being the highest due to its emergence in both round 2 of long washes (bar code AGG) and round 3 of long washes (bar code ACT). The other columns (in white) represent bar codes detected in the sequencing files, that are not used, that reflect possible low-level errors in misincorporation of a base in the synthesized primer and/or the amplification in subsequent PCR reactions and that appear therefore in next generation sequencing data. These sequences with errors in adaptors/barcodes (numbering 0.13%) were filtered out. We include these data as an example of the type of original data that can be acquired when this methodology is used. (B) Extracted Ubiquitin sequences were filtered to remove ‘background’ peptides and (C) Extracted NEDD8 sequences were filtered to remove ‘background’ peptides. These included peptides with three or more histidine residues since such peptides were defined as background-binding peptides to the nickel plate used in the selection. In addition, any peptides that were present more than one time in the parental library were also removed. Once such “background’ peptides were removed, the final list of peptides were acquired for Ubiquitin and NEDD8, respectively.

**Supplementary Table 3.** A list of the 60 peptides chosen for synthesis that bind either NEDD8, Ubiquitin, or both. Peptides were synthesized with a biotin tag at the N-terminus and examined for impacts on ubiquitin binding or ubiquitination. The list also contains mutated versions of key peptides such as those that derive from peptide-37 (Biotin- SGSGFIPAQLHFHWRS -NH2), including those with mutations (underlined) that are C-terminal to the “FIPA” motif, including; Biotin- SGSGFIPAQLHAHWRS -NH2, Biotin- SGSGFIPAQLHFHWWS -NH2, and Biotin- SGSGFIPAQLHFHRRS -NH2). These mutations in peptide-37 reduced binding of the peptide to Ubiquitin by ELISA (data not shown). This might suggest there is a high degree of specificity for peptide-37 that was specifically selected during phage-display.
